# Opening a new window on MR-based Electrical Properties Tomography with deep learning

**DOI:** 10.1038/s41598-019-45382-x

**Published:** 2019-06-20

**Authors:** Stefano Mandija, Ettore F. Meliadò, Niek R. F. Huttinga, Peter R. Luijten, Cornelis A. T. van den Berg

**Affiliations:** 1 Computational Imaging Group for MR diagnostic & therapy, Center for Image Sciences, University Medical Center Utrecht, Heidelberglaan 100, Utrecht, 3584 CX The Netherlands; 2 Department of Radiotherapy, Division of Imaging & Oncology, University Medical Center Utrecht, Heidelberglaan 100, Utrecht, 3584 CX The Netherlands; 3 Department of Radiology, University Medical Center Utrecht, Heidelberglaan 100, Utrecht, 3584 CX The Netherlands

**Keywords:** Magnetic resonance imaging, Magnetic resonance imaging

## Abstract

In the radiofrequency (RF) range, the electrical properties of tissues (EPs: conductivity and permittivity) are modulated by the ionic and water content, which change for pathological conditions. Information on tissues EPs can be used e.g. in oncology as a biomarker. The inability of MR-Electrical Properties Tomography techniques (MR-EPT) to accurately reconstruct tissue EPs by relating MR measurements of the transmit RF field to the EPs limits their clinical applicability. Instead of employing electromagnetic models posing strict requirements on the measured MRI quantities, we propose a data driven approach where the electrical properties reconstruction problem can be casted as a supervised deep learning task (DL-EPT). DL-EPT reconstructions for simulations and MR measurements at 3 Tesla on phantoms and human brains using a conditional generative adversarial network demonstrate high quality EPs reconstructions and greatly improved precision compared to conventional MR-EPT. The supervised learning approach leverages the strength of electromagnetic simulations, allowing circumvention of inaccessible MR electromagnetic quantities. Since DL-EPT is more noise-robust than MR-EPT, the requirements for MR acquisitions can be relaxed. This could be a major step forward to turn electrical properties tomography into a reliable biomarker where pathological conditions can be revealed and characterized by abnormalities in tissue electrical properties.

## Introduction

Non-invasive measurements of human tissue electrical properties (EPs), namely conductivity σ and relative permittivity ε_r_, are a challenge that attracted several research groups in the past decades^1,2^. These properties determine how electromagnetic (EM) fields, such as the MR radiofrequency fields (RF: 64–300 MHz), interact with human tissues. Tissue EPs depend on the tissue structure and composition (water content and ionic concentration). In particular, at RF frequencies where MRI works, tissue conductivity is modulated by the total ionic concentration, which varies in presence of pathologies. Several studies already showed a change in tissue conductivity in presence of tumors^3–8^. Therefore, non-invasive measurements of tissue EPs could in principle be used as a new biomarker in oncology for diagnostic purposes and treatment monitoring^7^.

The possibility to non-invasively measure tissue EPs at RF frequencies with clinical MRI systems was first suggested in the early 1990s^9^. However, systematic research only started in the last decade, creating a new branch of research called MR-Electrical Properties Tomography (MR-EPT)^10^. Using standard MR hardware, MR-EPT is able to reconstruct tissue EPs from measurements of the RF transmit magnetic field, i.e. the circularly polarized transverse magnetic field referred to as the ${\tilde{{\rm{B}}}}_{1}^{+}$ field. This field consists of incident and scattered field terms, where the latter component includes contributions from conduction and displacement currents, and thus contains the desired EPs information.

By applying the homogenous Helmholtz equation to the measured ${\tilde{{\rm{B}}}}_{1}^{+}$ field, EPs map can be reconstructed^9–11^. According to this analytical reconstruction model, tissue EPs maps can be obtained by computing second order spatial derivatives of the measured ${\tilde{{\rm{B}}}}_{1}^{+}$ field^11,12^. Spatial derivatives are computed by applying a filter (in this case a 2^nd^ order finite difference filter) to the ${\tilde{{\rm{B}}}}_{1}^{+}$ field data, resulting directly in EPs maps. However, this operation is highly sensitive to the intrinsic noise in the MR measurements, and consequently the reconstructed EPs maps lack precision^13,14^. To mitigate the impact of noise in the reconstructed EPs maps, large derivative filters in combination with image filters and large voxel sizes are commonly used^2^. Unfortunately, this comes at the cost of severe errors at tissue boundaries, thus making MR-EPT reconstructions challenging, especially for highly spatially convoluted tissue structures such as the human brain^14^. Furthermore, for clinical MRI systems (1.5 and 3 Tesla) permittivity reconstructions are not feasible, since the electromagnetic imprint of related displacement currents is too low at these frequencies.

Recently, alternative analytical reconstruction techniques have been presented to improve the quality of MR-EPT reconstructions^15–19^. However, these techniques require complex RF setups (multi-transmit array), and high field MR scanners (7 Tesla) are needed to achieve sufficient signal-to-noise-ratio (SNR). From a fundamental point of view, these analytical reconstruction techniques are attractive due to their direct forward mathematical formulation allowing fast reconstructions. However, these methods are sensitive to noise in the input data and therefore require relatively high SNR levels that are not always feasible at clinical MR field strengths.

To overcome this requirement, algebraic algorithms employing a more general inverse approach based on iterative minimization have been suggested^20–23^. These methods behave better under noisy conditions. However, this comes at the expense of a higher computational load, challenges related to local minima and more complex electromagnetic modeling. Moreover, these algebraic algorithms need a-priori information (e.g. incident MR electric field), which is not always available. Although some promising results from simulated data have been presented, accurate *in-vivo* reconstructions have not been shown yet.

Inspired by MR fingerprinting, a different reconstruction method called dictionary-based EPT has been recently proposed^24^. This method formulated the EPT reconstruction problem as a classification problem and it reconstructs tissue electrical conductivity on a 3D patch level by assigning the conductivity value that corresponds to the simulated ${\tilde{{\rm{B}}}}_{1}^{+}$ profile that best matched the measured ${\tilde{{\rm{B}}}}_{1}^{+}$ profile. First results showed the potential of such a matching approach for conductivity reconstructions. No permittivity reconstructions were presented yet. The presented methodology, which exploits a priori data, is not based on data driven learning strategy as in deep learning, where large amounts of realistic data is used to train neural networks.

Instead of relying on analytical or algebraic reconstruction techniques derived from electromagnetic theory, and given the potential of data driven approaches, in this work we investigate the feasibility of using a data driven, supervised deep learning (DL) approach for EPs reconstructions. Deep learning has recently been successfully applied to inverse problems including MRI image reconstruction^25–30^. To the best of our knowledge, this is the first time that deep learning is used for EPs reconstructions. Hereafter, we refer to this approach as Deep Learning Electrical Properties Tomography (DL-EPT).

Given the promising performance of Convolutional Neural Networks (CNNs), and in particular of Conditional Generative Adversarial Networks (cGANs)^31^, in this work we train a cGAN to perform EPs reconstructions. Contrary to state-of-the-art MR-EPT techniques which require electromagnetic quantities that are not directly accessible from MRI measurements (e.g. the phase of the MR transmit field, ${\tilde{\phi }}^{+}$), in DL-EPT a surrogate analytical reconstruction model can be learnt using only MR accessible quantities (e.g. the magnitude of the MR transmit field ${\tilde{{\rm{B}}}}_{1}^{+}$, and the transceive phase ${\tilde{\phi }}^{+}$). Electromagnetic simulations including realistic RF coil models, phantoms and body models are used to generate the training dataset. Nowadays, these datasets can be easily generated by exploiting the availability of sophisticated electromagnetic solvers, which allow realistic electromagnetic simulations (e.g. Sim4Life; CST; COMSOL; Remcom). In this way, a high degree of a-priori knowledge, such as the MRI coil setup, can be introduced.

In this work, DL-EPT reconstructions from simulations on phantoms and human head models as well as from phantom and *in-vivo* MR measurements at 3 Tesla using a clinically available MR setup are presented. The accuracy and precision of the reconstructed EPs maps are assessed, and the impact of different SNR levels has also been investigated. For comparison purposes, Helmholtz-based MR-EPT reconstructions (H-EPT) are presented as a reference for the phantoms and the head models simulations. Although the aim of this study is a proof of principle of DL-EPT, and not an investigation into optimal network and choice of learning parameters, several options are considered. In particular, two cGANs are employed: cGAN_mask_, and cGAN_tissue_. The former has in input the MR transit ${\tilde{{\rm{B}}}}_{1}^{+}\,\,$field magnitude, the phase ${\tilde{\phi }}^{+}$ (proportional to the transceive phase ${\mathop{\phi }\limits^{ \sim }}^{\pm }$ measurable in an MR experiment), and a binary mask (1: tissue, 0: air). In the latter, the binary mask is replaced by pseudo Spin Echo MRI images providing tissue contrast information. To the best of our knowledge, with this work we show for the first time that deep learning can provide improved reconstructions of electrical conductivity and permittivity using clinically available MRI scanners, coil setups, and realistic SNR levels.

## Results

**Figure 1:**
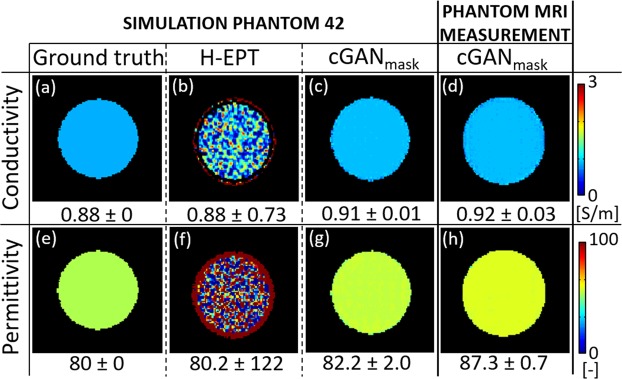
Conductivity and permittivity maps reconstructed using Helmholtz-based MR-EPT (H-EPT) (**b**,**f**) and cGAN_mask_ (**c**,**g**) for the phantom model 42. Ground truth EPs maps (**a**,**e**). cGAN_mask_ EPs reconstructions from MRI measurements at 3 Tesla (**d**,**h**). The reported numbers are the mean ± SD of the reconstructed EPs values inside a region of interest (see Supplementary Fig. S7).

In Fig. 1, H-EPT and DL-EPT reconstructions are presented for the phantom model 42 including realistic noise (see Supplementary Materials and Methods – Phantom and Head Models). This phantom was used for *in-silica* testing of the cGAN_mask_, and was not included in the training set. Additionally, reconstructions from MRI measurements at 3 T are presented for a cylindrical, homogeneous phantom with the same EPs values. The mean and standard deviation (SD) values of the reconstructed EPs maps are also reported in Fig. 1. To avoid boundary regions that cannot be reconstructed accurately in H-EPT, a smaller region of interest was considered for this evaluation (see Supplementary Fig. S7).

Phantom H-EPT reconstructions from simulated data show accurate mean EPs values after exclusion of boundary regions. However, the reported high SD values indicate lack of precision in the reconstructed EPs values due to severe noise amplification (see profiles in Supplementary Fig. S7). The need of high SNR levels is one of the main limitation of current analytical MR-EPT reconstruction methods.

On the contrary, DL-EPT reconstructions from simulated phantom data are less affected by noise. As reported in Fig. 1, DL-EPT reconstructions show a much better precision (low SD) at the cost of a small inaccuracy in the reconstructed mean EPs values (relative error <5%).

DL-EPT reconstructions from MR measurements confirm the results observed in simulations, thus demonstrating the feasibility of reconstructing EPs from MR measurements using DL-EPT. Additionally, permittivity reconstructions are now feasible at 3T, contrary to standard MR-EPT methods.

**Figure 2:**
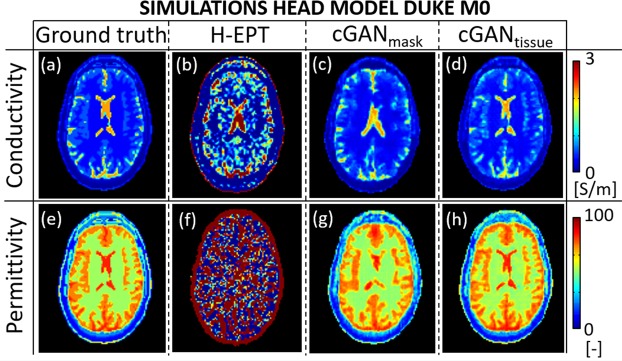
Head Model Duke M0 conductivity and permittivity reconstructions at 3 Tesla: (**a**,**e**) Ground truth, (**b**,**f**) H-EPT, (**c**,**g**) cGAN_mask_, (**d**,**h**) cGAN_tissue_.

In Fig. 2, H-EPT, cGAN_mask_, and cGAN_tissue_ EPs reconstructions are shown for the head model Duke M0, which was used for *in-silica* testing including noise. This head model was not included in the training set. Mean and standard deviation values for the white matter (WM), gray matter (GM), and cerebrospinal fluid (CSF) are reported in Table 1.

**Table 1:** Reconstructed EPs values for the Human Brain WM, GM and CSF.

	Conductivity σ [S/m]						Permittivity ε_r_ [−]					
	WM		GM		CSF		WM		GM		CSF	
	mean	(SD)	mean	(SD)	mean	(SD)	mean	(SD)	mean	(SD)	mean	(SD)
H-EPT Duke M0	0.33	(0.85)	0.64	(1.27)	3.22	(4.97)	52.9	(130)	67.8	(124)	−43	(350)
cGAN_mask_ Duke M0	0.34	(0.15)	0.56	(0.18)	1.83	(0.42)	52.5	(3.9)	72.9	(6.5)	84.1	(3.1)
cGAN_tissue_ Duke M0	0.34	(0.03)	0.60	(0.05)	2.03	(0.14)	53.1	(1.3)	74.3	(2.1)	84.4	(1.2)
cGAN_mask_ *in-vivo* subject 1	0.39	(0.08)	0.49	(0.16)	0.85	(0.48)	57.3	(7.2)	61.3	(7.9)	70.4	(10.0)
cGAN_tissue_ *in-vivo* subject 1	0.37	(0.04)	0.53	(0.18)	1.67	(0.47)	54.4	(3.2)	66.0	(6.9)	80.1	(4.9)
*Reference*	*0*.*34*	(*−*)	*0*.*59*	(−)	*2*.*14*	(−)	*52*.*6*	(−)	*73*.*4*	(−)	84	(−)

Mean and SD (inside brackets) of the reconstructed EPs values in the WM, GM, and CSF for the head model Duke M0 using H-EPT, cGAN_mask_, and cGAN_tissue_, and from *in-vivo* MR measurements on the first subject using cGAN_mask_, and cGAN_tissue_. A 3 voxels erosion was performed for each tissue type to avoid boundary regions, since these regions cannot be reconstructed accurately with H-EPT.

H-EPT conductivity reconstructions are severely affected by noise and boundary errors, as previously observed for the phantom reconstructions. Although average H-EPT conductivity and permittivity values for WM and GM have a relative error <10% with respect to input (ground truth) values after excluding boundary regions, the high standard deviations indicate that H-EPT is not suitable to reconstruct EPs on a voxel basis for highly spatially convoluted tissues.

If DL-EPT is used employing the cGAN_mask_, the precision of EPs reconstructions is greatly improved (much lower SD). If tissue contrast information (i.e. pseudo Spin Echo MRI images) is provided as additional input for the neural network (cGAN_tissue_), the precision of the reconstructed EPs maps is further improved, and the computed mean EPs values (Table 1) agree with the input (ground truth) values. As shown in Fig. 2 and Supplementary Fig. S10, the use of tissue contrast as a-priori information leads to less boundary errors, which are instead a major source of error in H-EPT reconstructions.

**Figure 3:**
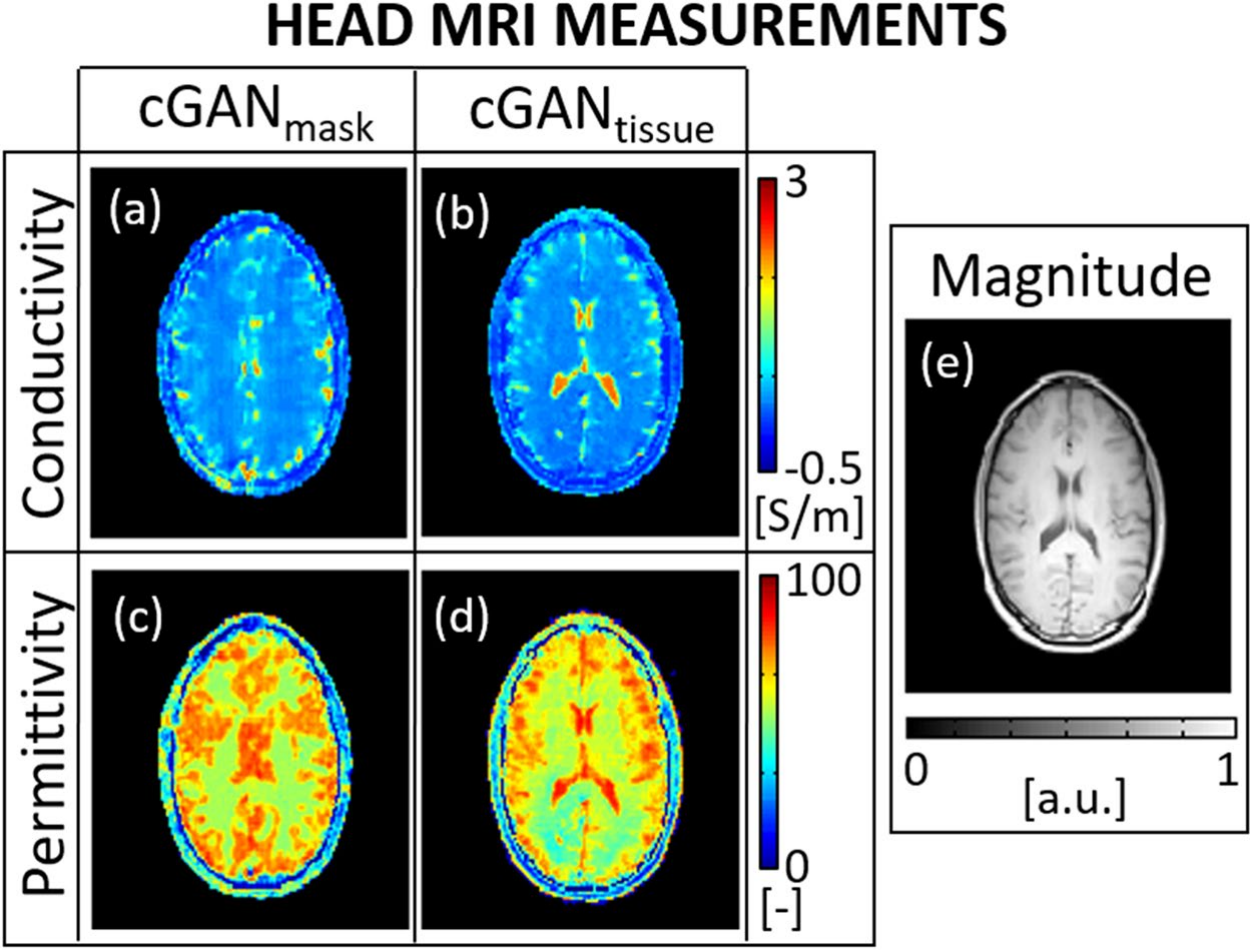
DL-EPT conductivity and permittivity reconstructions from MR measurements on the first subject using cGAN_mask_ and cGAN_tissue_ (**a**–**d**). The correspondent MRI magnitude image is also shown as a reference (**e**).

In Fig. 3, DL-EPT reconstructions from *in-vivo* MR measurements at 3T on a healthy subject are shown. Mean and standard deviation values are also reported in Table 1. DL-EPT reconstructions from other two healthy subjects are presented in the Supplementary Results – EPs Reconstructions (Supplementary Fig. S11 and Table S7). The presented results show good quality EPs maps ad exception for the head periphery and the ventricles where cGAN_mask_ demonstrates less performance. If tissue contrast information is provided, errors at tissue boundaries are considerably reduced. This confirms what was previously observed for the reconstructions from simulated data and shows the feasibility of using DL-EPT to reconstruct *in-vivo* EPs from MR measurements.

**Figure 4:**
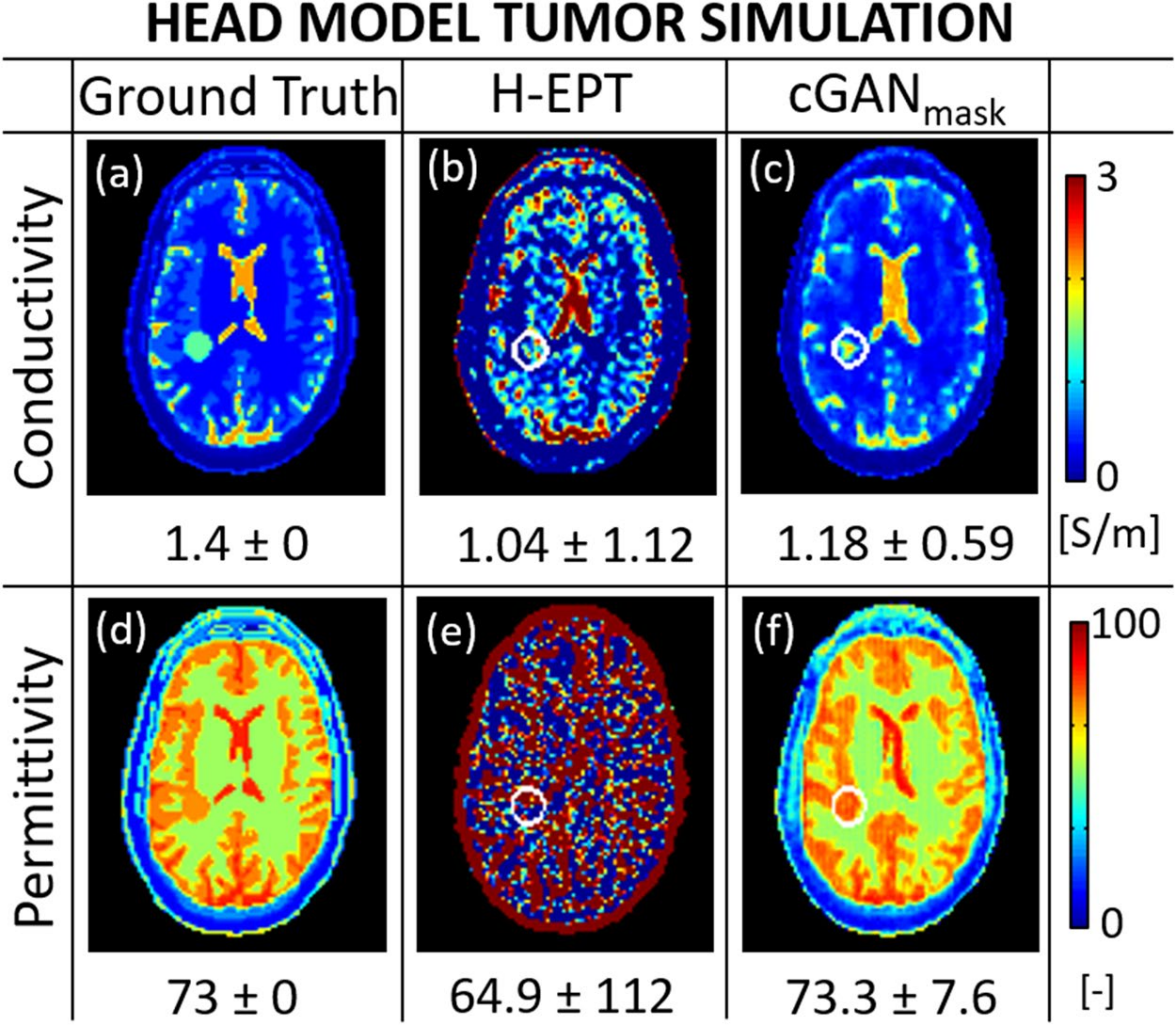
Ground truth EPs maps for Duke M0 with a tumor inclusion and H-EPT and cGAN_mask_ EPs reconstructions. The tumor contour is highlighted with a white circle in the reconstructed EPs maps. The numbers reported in the figure are the mean ± SD of the tumor EPs values.

Finally, in Fig. 4, a comparison between H-EPT and cGAN_mask_ EPs reconstructions for the head model Duke M0 with a tumor inclusion (sphere, radius 1.5 cm) is presented. Reconstructed mean EPs values and standard deviations of the tumor inclusion are also reported in the figure.

Correct identification of the tumor region is difficult for H-EPT reconstructions, which are highly corrupted by noise. Instead, cGAN_mask_ EPs reconstructions clearly show a tissue-tumor contrast, especially in the permittivity map. The presented DL-EPT reconstructions show an underestimation for the tumor conductivity value (relative error ≈ 15%), while the reconstructed tumor permittivity value is more accurate (relative error <5%). As a reference, DL-EPT reconstructions using the same network parameters and the same Duke model without tumor inclusion are reported in the Supplementary Materials (Supplementary Fig. S13).

## Discussion

In this work, a novel approach for EPs reconstructions is presented, namely deep learning electrical properties tomography (DL-EPT). This technique is based on a data driven learning task, where the training data are obtained from a large number of realistic electromagnetic simulations. We show for the first time that DL-EPT allows high quality conductivity and permittivity reconstructions of human brain tissues at clinically available MR field strengths using standard MR hardware. This has been investigated using *in-silica* data from realistic phantom and head models, as well as phantom and *in-vivo* MR measurements at 3 Tesla. The presented results show good accuracy and most notably precision in the reconstructed EPs maps on a voxel basis, demonstrating a large improvement with respect to MR-EPT techniques. Furthermore, DL-EPT is noise-robust and preserves boundary information, while these two aspects are the major issues for conventional MR-EPT techniques.

DL-EPT differs significantly from conventional MR-EPT techniques employing analytical or algebraic reconstruction models. The popular Helmholtz MR-EPT technique, an example of an analytical reconstruction technique, requires the computation of spatial derivatives on measured data^14^. This computation is performed by convolving the measured, complex ${\tilde{{\rm{B}}}}_{1}^{+}$ field with large finite difference kernels such as the 3D kernel adopted in this work^11^, or the Savitzky-Golay kernel^32^. These kernels, combined with image filters to further suppress the impact of noise^18,22^, lead to a much coarser effective resolution (order of 1 cm) and result in severe errors at tissue boundaries. On the other hand, algebraic MR-EPT reconstruction techniques employing iterative minimization, such as CSI-EPT^20^, should be more noise-robust. However, these methods require a large degree of regularization to stabilize noise augmentation in specific regions. Furthermore, these reconstruction techniques employ forward models formulated in electromagnetic quantities that are not always accessible with MRI, such as the phase of the transmit ${\tilde{{\rm{B}}}}_{1}^{+}$ MR field and the incident electric field. Currently, high quality experimental reconstructions using MR-EPT reconstructions are not yet available at clinical MRI field strengths (1.5 and 3 Tesla).

Given these limitations for MR-EPT reconstructions, we investigated the feasibility of using supervised deep learning to reconstruct EPs from accessible MR quantities. Crucial for the success of DL-EPT is the training part where a large degree of a-priori knowledge can be introduced by simulating a realistic coil setup and including realistic head models. The training requires a high number of unique complex ${\tilde{{\rm{B}}}}_{1}^{+}$ fields, which can be obtained by means of sophisticated, realistic electromagnetic simulations. Realistic electromagnetic simulations are nowadays possible with commonly available electromagnetic simulation software, and therefore represent an elegant solution to overcome the need of a high amount of MR data for training. Another fundamental advantage is that the DL-EPT reconstructions have additional flexibilities in the choice of the input parameters, i.e. the training can be performed based on quantities that are accessible with MRI measurements (e.g. transceive phase). This is contrary to conventional MR-EPT reconstructions models based on electromagnetic theory, which prescribes rigidly the required electromagnetic quantities that need to be measured.

Our results indicate that not only conductivity reconstructions at clinical MRI field strengths (1.5 and 3 Tesla) are feasible, but also permittivity maps can be obtained using DL-EPT. The latter were not yet feasible with conventional MR-EPT approaches due to insufficient SNR levels at clinical field strengths^13,33^. Preliminary investigations indicate that DL-EPT is more noise-robust than conventional MR-EPT reconstructions for SNR levels achievable in clinical MRI experiments (SNR ≈ 100). This is highly appealing, as it would permit to relax the requirements in terms of MRI data acquisition, allowing EPs measurements in clinical settings. Erroneous EPs reconstructions appear at SNR levels around 20 (see Supplementary Results – Impact of SNR). Low SNR values combined with CSF pulsation^34^ could be the cause of the observed inaccuracies at the periphery of the head and around the ventricles when cGAN_mask_ is used. More accurate EPs reconstructions at tissue boundaries can be obtained by including MR image information (tissue contrast) as a-priori knowledge. Future works should investigate whether other strategies are possible, e.g. providing the network with only boundary information instead of full tissue contrast information.

Of course, the use of a-priori knowledge during training could also create biased reconstructions for cases not included in the training phase. This would generally be the case for patients with pathologies. To test this risk, we provided the cGAN_mask_ with a pathological case that was not present in the training set, i.e. a head model including a brain tumor with altered EPs. In case of overfitting, which is a known issue for deep learning, reconstructions would not work anymore. Preliminary results at 3 Tesla seem to indicate that DL-EPT can provide a better tumor-normal tissue contrast than MR-EPT.

The presented results indicate the potential of DL-EPT for EPs reconstructions at clinical field strengths and standard MR coil setup. However, more studies are warranted to further validate and generalize DL-EPT.

It has to be further investigated what the impact of different learning parameters is on DL-EPT reconstructions and whether a single network can be trained to generalize to other field strengths and coil setups. We believe that a larger amount of diverse training data is needed for these purposes. Although optimum tuning of network parameters is beyond the scope of the current work, first results indicate that the quality of EPs reconstructions using cGAN_mask_ improve for different choices of learning parameters (Supplementary Fig. S13 and Table S9).

Additionally, it should be investigated whether the inclusion of *in-vivo* data during training would be beneficial to allow more accurate *in-vivo* EPs reconstructions. *In-vivo* measurements are affected by artifacts such as pulsation and motion, which are not present in simulations. MRI measurements of the ${\tilde{{\rm{B}}}}_{1}^{+}$ field are also corrupted by noise propagation and systematic errors which depend on the adopted ${\tilde{{\rm{B}}}}_{1}^{+}$ measurement technique^35^. These artifacts and variations in ${\tilde{{\rm{B}}}}_{1}^{+}$ fields may play a crucial role, resulting in less quality DL-EPT reconstructions from *in-vivo* MRI measurements compared to DL-EPT reconstructions from simulated data. For accurate *in-vivo* reconstructions, these artifacts may have to be included in simulations. Furthermore, it could be considered to include *in-vivo* EPT reconstructions in training data, even though the lack of ground truth EPs values for *in-vivo* cases might increase the level of complexity^36^.

Moreover, it will be fundamental to understand whether it will be necessary to include an exhaustive database of realistic pathological models (e.g. brain tumors) in the training set for accurate DL-EPT reconstructions of patients. We hypothesize that including more different training data might allow reducing the observed inaccuracies for cases not present in the training set. Future works should address these questions.

In this work, 2D DL-EPT reconstructions were performed due to the available network and computational power. However, given the 3D nature of the EPT reconstruction problem, the use of 3D neural networks for 3D DL-EPT reconstructions should be further studied, and the benefits of 3D patch-based approaches compared to image-based approaches, such as the one adopted in this work, should be addressed. We believe that 3D patch based approaches might allow better generalization of local features and less discontinuities in EPs reconstructions between slices.

In conclusion, to the best of our knowledge, this is the first demonstration of the feasibility of reconstructing *in-vivo* EPs from MR measurements using supervised deep learning. Although this work is a first proof of principle without aiming at identifying the best network architecture, which is beyond the current scope, the presented results indicate major improvements in the quality of the reconstructed EPs maps compared to MR-EPT approaches. Even permittivity reconstructions are now feasible at 3T with a standard and widely available coil setup. We showed that DL-EPT is noise-robust, thus the requirements in terms of SNR can be relaxed. This will allow faster imaging protocols and higher spatial resolutions. Moreover, DL-EPT can be trained with the transceive phase, thus circumventing the issue of measuring the ${\tilde{{\rm{B}}}}_{1}^{+}\,$phase, which is not directly accessible with MRI. The major finding of this work is that the application of supervised training for EPT reconstructions greatly improves the quality of the EP maps. This could have great impact in MR diagnostics as it would turn electrical properties mapping into a new reliable biomarker to locate and characterize pathological conditions based on differences in tissue ionic concentrations resulting in different tissue electrical properties.

## Materials and Methods

### Database construction

A database consisting of 42 homogeneous phantom models (diameter: 12 cm, length: 12 cm) and 20 head models with piecewise constant EPs values was created in Sim4Life (ZMT AG, Zurich, CH). Different EPs values were assigned to each phantom model and to the WM, GM, and CSF of the adopted head models (Duke and Ella, the Virtual Family^37^) (see Supplementary Materials and Methods – Phantom and Head Models). These models were placed inside a realistic birdcage body coil model resonant at 128 MHz, thus mimicking the experimental MR setup. With this setup, FDTD simulations were performed in Sim4Life to obtain realistic 3D ${\tilde{{\rm{B}}}}_{1}^{+}$ field magnitude and transceive phase (${\tilde{\phi }}^{+}$) maps. Thermal noise was included by independently adding Gaussian noise to the real and imaginary parts of the simulated fields (noiseless ${\tilde{{\rm{B}}}}_{1}^{+}\,$field magnitude and transceive phase).

The final SNR was 90 for the obtained ${\tilde{{\rm{B}}}}_{1}^{+}$ magnitude and the precision of the obtained phase ${\tilde{\phi }}^{+}$, proportional to the transceive phase, was 9 × 10^−3^ rad (see Supplementary Materials and Methods – Database Construction). This mimics realistic SNR levels in MR experiments. By means of these simulations, 2170 unique 2D complex ${\tilde{{\rm{B}}}}_{1}^{+}$ field distributions were generated (25 slices for each phantom model and 56 slices for each head model).

### Neural network

The neural network used for EPs reconstructions was a Conditional Generative Adversarial Network (cGAN)^31^. In this type of networks, two sub-networks (generator G, and discriminator D) compete with each other in a min-max optimization game during the training phase, in order to learn a conditional generative model. The generator network tries to generate EPs maps from the input images, while the discriminator network tries to discriminate the generated EPs maps from the EPs maps in the training set (ground truth). Like in Isola *et al*.^31^, the generator was a U-Net and the discriminator was a convolutional PatchGAN classifier. In Pathak *et al*.^38^, it was shown that using a cGAN combined with a L2 norm resulted in sharper images compared to a U-Net^39^. Afterwards, in Isola *et al*.^31^ it was demonstrated that the use of the L1 norm preserved the boundaries better in the reconstructed images. For EPs reconstructions, it is important to achieve good accuracy at tissue boundaries. Based on these observations, we combined a cGAN with both L1 and L2 norms, yielding to the following cost function (*F*):

1 $${\rm{F}}=\arg {min}_{{\rm{G}}}\,{max}_{{\rm{D}}}\,{\lambda }_{{\rm{c}}{\rm{G}}{\rm{A}}{\rm{N}}}{{\mathscr{L}}}_{{\rm{c}}{\rm{G}}{\rm{A}}{\rm{N}}}({\rm{G}},{\rm{D}})+{\lambda }_{{\rm{L}}1}{{\mathscr{L}}}_{{\rm{L}}1}({\rm{G}})+{\lambda }_{{\rm{L}}2}{{\mathscr{L}}}_{{\rm{L}}2}({\rm{G}}).$$

${ {\mathcal L} }_{cGAN}(G,D)$ is the GAN objective, ${ {\mathcal L} }_{L1}$ and ${ {\mathcal L} }_{L2}$ are respectively the L1 and L2 distance between the ground truth and the output, and λ_GAN_, λ_L1_, and λ_L2_ are the corresponding weights (see Supplementary Materials and Methods – Choice of cGAN).

This network was implemented in TensorFlow^40^ and trained in about four hours on a GPU (NVIDIA Tesla P100 16GB RAM). After training, 2D EPs reconstructions could be performed in less than 1 minute for a volume of 256 × 256 voxels in plane and 56 slices.

We first investigated the effect of providing the network only with EM quantities (cGAN_mask_). Then, we investigated the impact of providing the network with additional information, i.e. MRI tissue contrast (cGAN_tissue_). Although network optimization is beyond the scope of this work, we also investigated the impact of few different learning parameters on DL-EPT reconstructions for cGAN_mask_ (Supplementary Fig. S13).

### DL-EPT: cGAN_mask_

For the training, 2014 2D complex ${\tilde{{\rm{B}}}}_{1}^{+}$ field distributions were generated using all the simulated models, except for the phantom models 12, 24, 38, and 42, and the head model Duke M0. The inputs for the neural network were: the ${\tilde{{\rm{B}}}}_{1}^{+}$ magnitude, the phase ${\tilde{\phi }}^{+}$ (proportional to the transceive phase), and a binary mask (1: object, 0: air). Since only a binary mask was provided as third input and not information about tissue structure, we define this network as cGAN_mask_. To reduce the complexity of the problem, two networks with the same input data were trained separately for conductivity and permittivity reconstructions using the same combinations of λ-weights.

For the validation, the complex ${\tilde{{\rm{B}}}}_{1}^{+}$ field distributions of the phantom models 12 and 24 were used. Although the aim of the paper was not to find the best combination of λ_GAN_, λ_L1_, and λ_L2_ weights, we investigated the impact of various combinations of these parameters on the reconstructed EPs maps. The parameters combination with the lowest average normalized-root-mean-square error (NRMSE) computed over the conductivity and permittivity reconstructions from the validation set was selected for testing: λ_GAN_ = 2, λ_L1_ = 100, and λ_L2_ = 200 (see Supplementary Materials and Methods – Choice of cGAN). Among the combinations tested, we investigated whether setting λ_GAN_ = 0, i.e. employing a less sophisticated network (U-Net)^39^, would be sufficient for EPs reconstructions (see Supplementary Results – Comparison U-Net and cGAN_mask_).

For testing of the selected cGAN_mask_, the complex ${\tilde{{\rm{B}}}}_{1}^{+}$ field distributions of the phantom models 38 and 42, and Duke model M0 were used. The performed realistic electromagnetic simulations provide a controlled environment in which knowledge of the ground truth, i.e. conductivity and permittivity, is possible. This ensured correct assessment of the accuracy (absolute errors: ∆σ, and ∆ε_r_) and precision (standard deviation SD) of the performed EPs reconstructions. Additionally, this network was tested using phantom an *in-vivo* MR measurements. The adopted phantom was a homogeneous, agar-based phantom: diameter: 13 cm, length: 15 cm, σ: 0.88 S/m; ε_r_: 80, obtained from probe measurements at 21 °C (85070E, Agilent Technologies, Santa Clara, CA, USA). *In-vivo* MR measurements were performed on three healthy subjects (male, mean age 26, SD 2.6), after obtaining written informed consent. This was approved by the local institutional review board of the University Medical Center Utrecht, and carried out in accordance with the relevant guidelines and regulations.

Furthermore, to test the generalizability, we investigated the feasibility of detecting a tumor without having trained the neural network with brain tumor models and without providing any information on tissue structure. For this purpose, a head tumor model was created by placing one sphere inside Duke M0 (radius 1.5 cm, σ: 1.4 S/m; ε_r_: 73). For this test, the parameter combination with the lowest average NRMSE value computed over conductivity and permittivity reconstructions in the WM, GM and CSF of Duke M0 was chosen: λ_GAN_ = 2, λ_L1_ = 1000, and λ_L2_ = 2000 (see Supplementary Table S9). DL-EPT reconstructions for Duke M0 using these network parameters are shown as a reference in the supplementary materials (Supplementary Fig. S13).

### DL-EPT: cGAN_tissue_

Since MRI images show good contrast between different tissues, we investigated whether providing tissue contrast information as third input instead of adopting a simple mask would improve the EPs reconstructions for the human brain. We therefore trained a cGAN using only the 1064 2D complex ${\tilde{{\rm{B}}}}_{1}^{+}$ field distributions of the brain models (except for Duke M0, which was used for testing) and the combination of λ-weights previously chosen for the brain reconstructions from simulations and MR measurements, thus allowing direct comparison with the results obtained using the cGAN_mask_. Hence, the inputs were: the ${\tilde{{\rm{B}}}}_{1}^{+}$ magnitude, the phase ${\tilde{\phi }}^{+}$ and pseudo Spin echo magnitude images obtained after assigning to each tissue type the corresponding magnitude value that would be measured in those tissues with a Spin Echo sequence (see Supplementary Materials and Methods –MR Sequences). We define this network as cGAN_tissue_, since the third input provides tissue contrast information. This network was tested on Duke M0 and *in-vivo* MRI data.

### MRI measurements

MRI measurements were performed with a 3 Tesla MR scanner (Ingenia, Philips HealthCare, Best, The Netherlands) with the body coil in transmit and a 15-channel head coil in receive mode. The ${\tilde{{\rm{B}}}}_{1}^{+}$ magnitude was measured using a dual-TR (AFI) sequence^41^. To map the transceive phase, two single echo Spin Echo (SE) sequences with opposite readout gradient polarities were combined^11^: (ϕ_SE1_ − ϕ_SE2_)/2, thus minimizing the impact of eddy-currents related artifacts. To convert the receive phase measured with the head coil to the body coil, as if the body coil would have been used both for transmitting and receiving, the vendor specific algorithm CLEAR (Constant Level of Appearance) was used. The sequence parameters for the phantom and the *in-vivo* MRI measurements are reported in Supplementary Materials and Methods – MR Sequences.

### MR-EPT reconstructions: H-EPT

For comparison purposes, standard Helmholtz-based MR-EPT reconstructions (H-EPT) were also performed for the simulated phantom models 38 (see Supplementary Results – EPs Reconstructions) and 42, and the head model Duke M0 with and without tumor inclusion according to^11^:

2 $${{\rm{\varepsilon }}}_{{\rm{r}}}({\bf{r}})=\frac{-1}{{{\rm{\mu }}}_{0}{{\rm{\varepsilon }}}_{0}{{\rm{\omega }}}^{2}}{\rm{Re}}(\frac{{\nabla }^{2}{\tilde{{\rm{B}}}}_{1}^{+}({\bf{r}})}{{\tilde{{\rm{B}}}}_{1}^{+}({\bf{r}})})$$

3 $${\rm{\sigma }}({\bf{r}})=\frac{1}{{{\rm{\mu }}}_{0}{\rm{\omega }}}{\rm{Im}}(\frac{{\nabla }^{2}{\tilde{{\rm{B}}}}_{1}^{+}({\bf{r}})}{{\tilde{{\rm{B}}}}_{1}^{+}({\bf{r}})})$$

with *ω*: Larmor angular frequency, *ε*_0_/*μ*_0_: free space permittivity/permeability, and ***r***: x/y/z-coordinates. To compute the second order spatial derivatives, a 3D noise-robust kernel was used (7 × 7 × 5 voxels)^11^.

## Data Availability

Data are available from the corresponding author upon reasonable request.

## Supplementary information

Supplementary Information

## References

[1] Katscher Ulrich, Kim Dong-Hyun, Seo Jin Keun (2013). Recent Progress and Future Challenges in MR Electric Properties Tomography. Computational and Mathematical Methods in Medicine.

[2] Katscher U, van den Berg CAT (2017). Electric properties tomography: Biochemical, physical and technical background, evaluation and clinical applications. NMR Biomed..

[3] van Lier, A. L. H. M. W. *et al*. Electrical conductivity imaging of brain tumours. Proc. *19th Annu. Meet. ISMRM*. Montréal, Québec, Canada 4464 (2011).

[4] Katscher, U. *et al*. Estimation of breast tumor conductivity using parabolic phase fitting. *Proc. 20th Sci. Meet. Int. Soc. Magn. Reson. Med*. Melbourne, Victoria, Aust. 2335 (2012).

[5] Shin J (2015). Initial study on *in vivo* conductivity mapping of breast cancer using MRI. J. Magn. Reson. Imaging.

[6] Balidemaj E (2016). *In vivo* electric conductivity of cervical cancer patients based on B1+ maps at 3T MRI. Phys. Med. Biol..

[7] Kim SY (2016). Correlation between conductivity and prognostic factors in invasive breast cancer using magnetic resonance electric properties tomography (MREPT). Eur. Radiol..

[8] Tha Khin Khin, Katscher Ulrich, Yamaguchi Shigeru, Stehning Christian, Terasaka Shunsuke, Fujima Noriyuki, Kudo Kohsuke, Kazumata Ken, Yamamoto Toru, Van Cauteren Marc, Shirato Hiroki (2017). Noninvasive electrical conductivity measurement by MRI: a test of its validity and the electrical conductivity characteristics of glioma. European Radiology.

[9] Haacke EM, Petropoulos LS, Nilges EW, Wu DH (1991). Extraction of conductivity and permittivity using magnetic resonance imaging. Phys. Med. Biol..

[10] Katscher U (2009). Determination of Electric Conductivity and Local SAR Via B1 Mapping. IEEE Trans. Med. Imaging.

[11] van Lier ALHMW (2012). B1+ phase mapping at 7 T and its application for *in vivo* electrical conductivity mapping. Magn. Reson. Med..

[12] van Lier ALHMW (2014). Electric properties tomography in the human brain at 1.5, 3, and 7 T: a comparison study. Magn. Reson. Med..

[13] Lee S, Bulumulla S, Hancu I (2015). Theoretical Investigation of Random Noise-Limited Signal-to-Noise Ratio in MR-Based Electrical Properties Tomography. IEEE Trans. Med. Imaging.

[14] Mandija S, Sbrizzi A, Katscher U, Luijten PR, van den Berg CAT (2018). van den Berg, C. a. T. Error analysis of Helmholtz-based MR-electrical properties tomography. Magn. Reson. Med..

[15] Sodickson, D. K. *et al*. Generalized Local Maxwell Tomography for Mapping of Electrical Property Gradients and Tensors. *Proc. 21st Sci. Meet. Int. Soc. Magn. Reson. Med*. Salt Lake City, Utah, USA 4175 (2013).

[16] Hafalir FS, Oran OF, Gurler N, Ider YZ (2014). Convection-reaction equation based magnetic resonance electrical properties tomography (cr-MREPT). IEEE Trans. Med. Imaging.

[17] Liu J, Zhang X, Schmitter S, Van de Moortele P-F, He B (2015). Gradient-based electrical properties tomography (gEPT): A robust method for mapping electrical properties of biological tissues *in vivo* using magnetic resonance imaging. Magn. Reson. Med..

[18] Gurler N, Ider YZ (2016). Gradient-Based Electrical Conductivity Imaging Using MR Phase. Magn. Reson. Med..

[19] Marques JP, Sodickson DK, Ipek O, Collins CM, Gruetter R (2015). Single acquisition electrical property mapping based on relative coil sensitivities: A proof-of-concept demonstration. Magn. Reson. Med..

[20] Balidemaj E (2015). CSI-EPT: A Contrast Source Inversion Approach for Improved MRI-Based Electric Properties Tomography. IEEE Trans. Med. Imaging.

[21] Borsic A, Perreard I, Mahara A, Halter RJ (2016). An inverse problems approach to MR-EPT image reconstruction. IEEE Trans. Med. Imaging.

[22] Ropella KM, Noll DC (2017). A regularized, model-based approach to phase-based conductivity mapping using MRI. Magn. Reson. Med..

[23] Serralles, J. E. *et al*. Global Maxwell Tomography: A novel technique for electrical properties mapping without symmetry assumption or edge artifacts. *Proc. 24th Sci. Meet. Int. Soc. Magn. Reson. Med*. Singapore 2993 (2016).

[24] Hampe N (2019). Dictionary-based electric properties tomography. Magn. Reson. Med..

[25] Golkov Vladimir, Dosovitskiy Alexey, Sämann Philipp, Sperl Jonathan I., Sprenger Tim, Czisch Michael, Menzel Marion I., Gómez Pedro A., Haase Axel, Brox Thomas, Cremers Daniel (2015). q-Space Deep Learning for Twelve-Fold Shorter and Model-Free Diffusion MRI Scans. Lecture Notes in Computer Science.

[26] Hammernik K (2018). Learning a variational network for reconstruction of accelerated MRI data. Magn. Reson. Med..

[27] Işin A, Direkoǧlu C, Şah M (2016). Review of MRI-based Brain Tumor Image Segmentation Using Deep Learning Methods. Procedia Comput. Sci..

[28] Hyun, C. M., Kim, H. P., Lee, S. M., Lee, S. & Seo, J. K. Deep learning for undersampled MRI reconstruction. arXiv 1709.02576, 1–11 (2017). 10.1088/1361-6560/aac71a 29787383

[29] Zhu B, Liu JZ, Rosen BR, Rosen MS (2018). Image reconstruction by domain transform manifold learning. Nature.

[30] Maspero, M. *et al*. Fast synthetic CT generation with deep learning for general pelvis MR-only Radiotherapy. arXiv 1802.06468, 1–14 (2018). 10.1088/1361-6560/aada6d 30109989

[31] Isola, P., Zhu, J.-Y., Zhou, T. & Efros, A. A. Image-to-Image Translation with Conditional Adversarial Networks. arXiv 1611.07004, 1–16 (2016).

[32] Savitzky A, Golay MJE (1964). Smoothing and Differentiation of Data by Simplified Least Squares Procedures. Anal. Chem..

[33] Duan S (2016). Quantitative analysis of the reconstruction errors of the currently popular algorithm of magnetic resonance electrical property tomography at the interfaces of adjacent tissues. NMR Biomed..

[34] Katscher, U., Stehning, C. & Tha, K. K. The impact of CSF pulsation on reconstructed brain conductivity. *Proc. 26th Sci. Meet. Int. Soc. Magn. Reson. Med*. Paris 546 (2018).

[35] Gavazzi S (2019). Accuracy and precision of electrical permittivity mapping at 3T: the impact of three B1+ mapping techniques. Magn. Reson. Med..

[36] Hampe, N., Katscher, U., van den Berg, C. A. T. & Mandija, S. Deep learning brain conductivity mapping using a patch-based 3D U-net. *Proc. 27th Sci. Meet. Int. Soc. Magn. Reson. Med*., Montreal, Canada, 5045 (2019).

[37] Christ A (2010). The Virtual Family - Development of surface-based anatomical models of two adults and two children for dosimetric simulations. Phys. Med. Biol..

[38] Pathak, D., Krahenbuhl, P., Donahue, J., Darrell, T. & Efros, A. a. Context Encoders: Feature Learning by Inpainting. arXiv 1604.07379, 12 (2016).

[39] Ronneberger, O., Fischer, P. & Brox, T. U-Net: Convolutional Networks for Biomedical Image Segmentation. arXiv 1505.04597, 234–241 (2015).

[40] Abadi, M. *et al*. TensorFlow: Large-Scale Machine Learning on Heterogeneous Distributed Systems. arXiv 1603.04467, 1–19 (2016).

[41] Yarnykh VL (2007). Actual flip-angle imaging in the pulsed steady state: A method for rapid three-dimensional mapping of the transmitted radiofrequency field. Magn. Reson. Med..

