# Supplementary material for: Opening a new window on MR-based Electrical Properties Tomography with deep learning
Source: Sci Rep. 2019 Jun 20;9:8895. doi: 10.1038/s41598-019-45382-x (PMC6586684; doi:10.1038/s41598-019-45382-x)
Supplement: Supplementary file 1 — Supplementary Information [file 41598_2019_45382_MOESM1_ESM.pdf]

Supplementary Information for:

Opening a new window on MR-based Electrical Properties Tomography with deep learning.

Stefano Mandija*, Ettore F. Meliadò, Niek R.F. Huttinga, Peter R. Luijten, Cornelis A.T. van den Berg.

This file includes:

Supplementary Information Text. 2

Supplementary Figures S1 to S15. 5

Supplementary Tables S1 to S9. 15

Supplementary Information Text

Supplementary Materials and Methods

Phantom and Head Models. 42 cylindrical phantom models and the 20 head models were created in Sim4Life (ZMT AG, Zurich, CH). The ground truth EPs values of these models are reported in the Supplementary Tables S1 and S2, respectively. In order to introduce more variability between the adopted head models, not only the conductivity and permittivity values of WM, GM and CSF were changed between models, but also geometrical transformations were applied with respect to the original models (Duke M0 and Ella M0)[1]. These transformations include compression/dilatation of the head models, as well as rotation and translation, thus mimicking different possible head orientations inside the MR bore. For each head model, ground truth EPs maps are shown for one slice (red plane, Supplementary Figs S1 and S2). This slice was taken on the same plane for all the head models with respect to the considered volume of interest (yellow box). Therefore, the observed variability between subfigures is due to the performed geometrical transformations and variations in the EPs for the simulated head models.

Database Construction.
Two simulations were performed in Sim4Life for each phantom and head model (Supplementary Fig. S3): one in quadrature mode (QA), and one in anti-quadrature mode (AQ). Contrary to conventional MR-EPT approaches, which reconstruction models require the non-measurable RF transmit phase φ^+ (approximated with $\frac{\varphi^\pm}{2}$: the so-called transceive phase assumption)[2], here the transceive phase ($\varphi^\pm$) was used, i.e. the phase measurable in an MR experiment. From these simulations, the electromagnetic quantity $\widehat{B}_1^+$ was obtained (Supplementary Fig. S4). $\widehat{B}_1^+$ consists of the transmit B_1^+ field magnitude and the phase $\widehat{\varphi}^+$ proportional to the transceive phase: $\widehat{\varphi}^+ = (\frac{\varphi^\pm}{2}) = \frac{\varphi^+ + \varphi^-}{2}$, where $\widehat{\varphi}^+ \neq \varphi^+$ since $\varphi^+ \neq \varphi^-$. Then, Gaussian noise was independently added to the real and imaginary parts of the computed complex $\widehat{B}_1^+$ field. Finally, the magnitude and the phase of the obtained noise-corrupted $\widetilde{B}_1^+$ fields were used as inputs for the cGANs (Supplementary Fig. S4). The SNR of $\left|\widetilde{B}_1^+\right|$ maps and the precision of $\widetilde{\varphi}^+$maps obtained from the simulations were defined as:

$$\mathrm{SNR}_{|\widetilde{B}_1^+|} = \frac{\mathrm{mean}(|\widetilde{B}_1^+|)}{\mathrm{std}(|\widetilde{B}_1^+| - |B_1^+|)},$$

$$\Delta\widetilde{\varphi}^+ = \frac{1}{\mathrm{SNR}_{|\widetilde{B}_1^+|}}.$$

To reduce the complexity of the reconstruction problem, cGANs were independently trained for permittivity and conductivity reconstructions, but the same values were used for the network weights λ_{GAN}, λ_{L1}, and λ_{L2}. The inputs were the magnitude of the noise-corrupted $\widetilde{B}_1^+$ field, the phase $\widetilde{\varphi}^+$ (proportional to the transceive phase $\widetilde{\varphi}^\pm$ measurable in an MR experiment) and a binary mask (1 for tissue and 0 for air). We define this network as cGAN_{mask}. To investigate the impact of tissue information on the accuracy of the reconstructed EPs values, pseudo Spin Echo images were used instead of the binary mask as third input. We define this network as cGAN_{tissue} (Supplementary Fig. S5). These pseudo Spin Echo images were created for each brain model as it follows. First, reference magnitude values were computed for each brain tissue from MRI measurements on a healthy subject performed using a Spin Echo sequence (see Supplementary Materials and Methods – MR Sequences). In particular, these reference values are mean magnitude values computed for each tissue type inside regions with a homogeneous $\widetilde{B}_1^+$ magnitude field distribution. These values were applied to the corresponding tissue type of each brain model. Then, the obtained maps were scaled using the simulated B_1^+ magnitude field distribution for each head model. Finally, Gaussian noise was added using the same SNR level adopted for the phase maps $\widetilde{\varphi}^+$.
For comparison purposes, one slice of the acquired MRI Spin Echo images on a healthy subject and one slice of the computed pseudo Spin Echo maps for Duke Model M0 are shown in the Supplementary Fig. S6. Mean values computed in different ROIs show good agreement between the MRI Spin Echo image and the pseudo Spin Echo image (Supplementary Table S3).

Choice of cGAN$_{mask}$. Formally, $\mathcal{L}_{cGAN}$, $\mathcal{L}_{L1}$ and $\mathcal{L}_{L2}$ are defined as:

$$\mathcal{L}_{cGAN} = \mathbb{E}_{x,y\sim p_{data}(x,y)}[\log D(x,y)] + \mathbb{E}_{x\sim p_{data}(x),z\sim p_z(z)}\left[\log\left(1 - D\big(x, G(x,z)\big)\right)\right]$$

$$\mathcal{L}_{L1} = \mathbb{E}_{x,y\sim p_{data}(x,y),z\sim p_z(z)}[\|y - G(x,z)\|_1]$$

$$\mathcal{L}_{L2} = \mathbb{E}_{x,y\sim p_{data}(x,y),z\sim p_z(z)}[\|y - G(x,z)\|_2],$$

where x represents $\left\{\left|\widetilde{B}_1^+\right|, \widetilde{\varphi}^+, mask\right\}$ or $\left\{\left|\widetilde{B}_1^+\right|, \widetilde{\varphi}^+, pseudo\ MRI\right\}$ in the training set, y are the corresponding ground truth EPs maps and z is a vector drawn from the probability distribution p_z[3]. As reported in Isola[3], p_z is ignored by the network and therefore it is left out in the network implementation.
Different weights (λ_{GAN}, λ_{L1}, and λ_{L2}) were used during training. The phantom models 12 and 24, which were excluded from the training set, were used in the validation step to choose which combination of λ-weights had the lowest average normalized-root-mean-square error (NRMSE) computed over the reconstructed EPs values of both phantoms. This combination of λ-weights was: $\lambda_{GAN} = 2$, $\lambda_{L1} = 100$, and $\lambda_{L2} = 200$ (Supplementary Table S4). This combination was therefore used for testing using the phantom models 38, and 42, the phantom MRI measurements, the head model Duke M0 and the *in-vivo* MRI measurements. Of course, the phantom and head models, as well as the phantom and *in-vivo* MRI measurements used for the validation and the testing steps were excluded from the training dataset.

MR Sequences. In the Supplementary Tables S5 and S6 are reported the MR sequence parameters used for the Actual Flip Angle Imaging (AFI) sequence and for the two Spin Echo sequences acquired with opposite readout gradient polarities. From the AFI sequence, $\widetilde{B}_1^+$ magnitude maps were obtained[4]. From the Spin Echo sequences, $\widetilde{\varphi}^+$ maps were computed[5].

H-EPT Reconstructions: For completeness and comparison purposes, noiseless H-EPT reconstructions for Duke M0 using the large 3D noise-robust kernel (7×7×5voxels) and a minimal kernel (3×3×3voxels) are presented.

Supplementary Results

EPs Reconstructions. In the Supplementary Fig. S7, the profiles of the reconstructed conductivity and permittivity maps for the phantom model 42 using H-EPT (blue) and cGAN$_{mask}$ (red) are shown. These profiles were taken in direction left/right, as shown in the subfigures on the right (black lines). In these subfigures, the gray circles indicate the region of interest (ROI) used to compute the mean and SD of the reconstructed EPs values for the phantom models used for validation (phantom models 12, and 24) and for testing (phantom models 38, 42, and phantom MR measurements). The same ROI was used for all the other slices of the phantoms. In this way, errors arising from boundary regions in H-EPT reconstructions were excluded.
In the Supplementary Fig. S8, the absolute error maps of conductivity and permittivity reconstructions are shown for the phantom model 42 and for the phantom MR measurements, which were used for testing of the selected cGAN$_{mask}$. The absolute error for conductivity reconstructions is below 0.05 S/m (less than 5% relative error), for both the simulation and the MR measurement. The absolute error for permittivity reconstructions is below 5 for the simulated data, while it is a bit higher (about 8) for the reconstruction from the MR measurement. The higher error in permittivity reconstructions from MR measurements can be explained by intrinsic inaccuracies in the adopted $\widetilde{B}_1^+$ magnitude mapping technique. The absolute error for H-EPT reconstructions from simulated data is instead one order of magnitude higher than the error observed for the cGAN$_{mask}$ reconstructions.
In the Supplementary Fig. S9, the reconstructed EPs maps for the phantom model 38, which was also used for testing, and the mean ± SD of the reconstructed EPs values are reported. The relative errors for these reconstructions are in line with the relative errors previously observed for the phantom model 42.
In the Supplementary Fig. S10, absolute error maps for conductivity and permittivity reconstructions for the head model Duke M0 are presented. From these maps, it can be observed that the absolute error at tissue boundaries can be

reduced if tissue information is given in input to the cGAN. In contrast, the absolute error for H-EPT reconstructions is at least one order of magnitude higher than the errors reported for the adopted cGANs.
In the Supplementary Fig. S11, *in-vivo* DL-EPT reconstructions for the second and the third subject are shown. The mean and SD values of the reconstructed EPs in the WM, GM, and CSF are reported in Supplementary Table S7. These results confirm what was previously observed in the main manuscript for the first subject, thus showing the feasibility of reconstructing tissue EPs *in-vivo* using DL-EPT.

Impact of SNR. The impact of different SNR levels (no noise, 50, 20, and 5) on EPs reconstructions was investigated for the selected $cGAN_{mask}$ using the head model Duke M0. From the Supplementary Fig. S12 and Table S8, it is visible that only for low SNR levels (less than 20) EPs reconstructions are not accurate anymore. Typical SNR levels in MR experiments are higher than this value, thus suggesting that deep learning approaches would be sufficiently noise-robust for EPs reconstructions from MR measurements. Still, adequate knowledge on the SNR limits for DL-EPT reconstructions would be fundamental to allow for faster MR sequences with higher spatial resolutions (voxel size in the order of 1 mm) than typically employed MR sequences for EPs reconstructions.

Comparison U-Net and cGANs. To investigate the impact of different λ-weights on the reconstructed DL-EPT values of brain tissues, the average NRMSE was computed over the reconstructed EPs values in the WM, GM, and CSF of Duke Model M0. From the Supplementary Table S9, it can be observed that the combination of λ-weights giving the lowest average NRMSE for $cGAN_{mask}$ is: $\lambda_{GAN} = 2$, $\lambda_{L1} = 1000$, and $\lambda_{L2} = 2000$. This $cGAN_{mask}$ was used for DL-EPT reconstructions on the Duke model M0 with a tumor inclusion. It can also be observed that setting $\lambda_{GAN} = 0$, thus using a U-Net instead of a cGAN, could in principle lead to accurate results. For sake of completeness, a comparison between EPs reconstructions for Duke M0 using the U-net, and the cGANs adopted in the manuscript is presented in Supplementary Fig. S13.
From the Supplementary Fig. S13, it appears that EPs reconstructions using a U-Net are more blurred than cGANs reconstructions. However, we do not exclude that different training parameters and more exhaustive training sets could allow more accurate reconstructions at tissue boundaries. This will be focus of future works.

H-EPT Reconstructions: These reconstructions demonstrate that H-EPT provides accurate EPs reconstructions only in large homogeneous regions for noiseless cases (Supplementary Fig. S14). However, even if a small kernel is used, severe errors at tissue boundaries are observed. For real cases with the presence of noise, large kernels need to be employed in H-EPT for noise robust reconstructions, however, at the cost of a larger spatial extension of boundary errors. For the SNR level adopted in this manuscript, which is typical for an MRI experiment, H-EPT conductivity reconstructions are of poor quality and permittivity reconstructions are not feasible. This is due to presence of boundary errors as well as errors due to noise amplification introduced by the numerical Laplacian operation[5].

$cGAN_{tissue}$ rescaling: To test whether $cGAN_{tissue}$ would learn a rescaling using only the pseudo Spin Echo image and discarding the transceive phase and the magnitude of the B_1^+, we gave as an input to the $cGAN_{tissue}$ network only the pseudo Spin Echo images of Duke M0. If $cGAN_{tissue}$ output would rely heavily on the pseudo Spin Echo image intensity and learn a simple rescaling for EPs maps generation, we would expect that the $cGAN_{tissue}$ output should still be EPs maps. However, as shown in the Supplementary Fig. S15, this is not the case, indicating that B_1^+ magnitude and phase information are needed. Future work should investigate whether other strategies are possible, e.g. providing only boundary information instead of full tissue information.

Supplementary References

1. Christ, A. *et al.* The Virtual Family - Development of surface-based anatomical models of two adults and two children for dosimetric simulations. *Phys. Med. Biol.* **55,** 23–38 (2010).
2. van Lier, A. L. H. M. W. *et al.* B1+ phase mapping at 7 T and its application for in vivo electrical conductivity mapping. *Magn. Reson. Med.* **67,** 552–561 (2012).
3. Isola, P., Zhu, J.-Y., Zhou, T. & Efros, A. A. Image-to-Image Translation with Conditional Adversarial Networks. https://arxiv.org/abs/1611.07004 (2016).
4. Yarnykh, V. L. Actual flip-angle imaging in the pulsed steady state: A method for rapid three-dimensional mapping of the transmitted radiofrequency field. *Magn. Reson. Med.* **57,** 192–200 (2007).
5. Mandija, S., Sbrizzi, A., Katscher, U., Luijten, P. R. & van den Berg, C. a. T. Error analysis of helmholtz-based MR-electrical properties tomography. *Magn. Reson. Med.* **80,** 90–100 (2018).

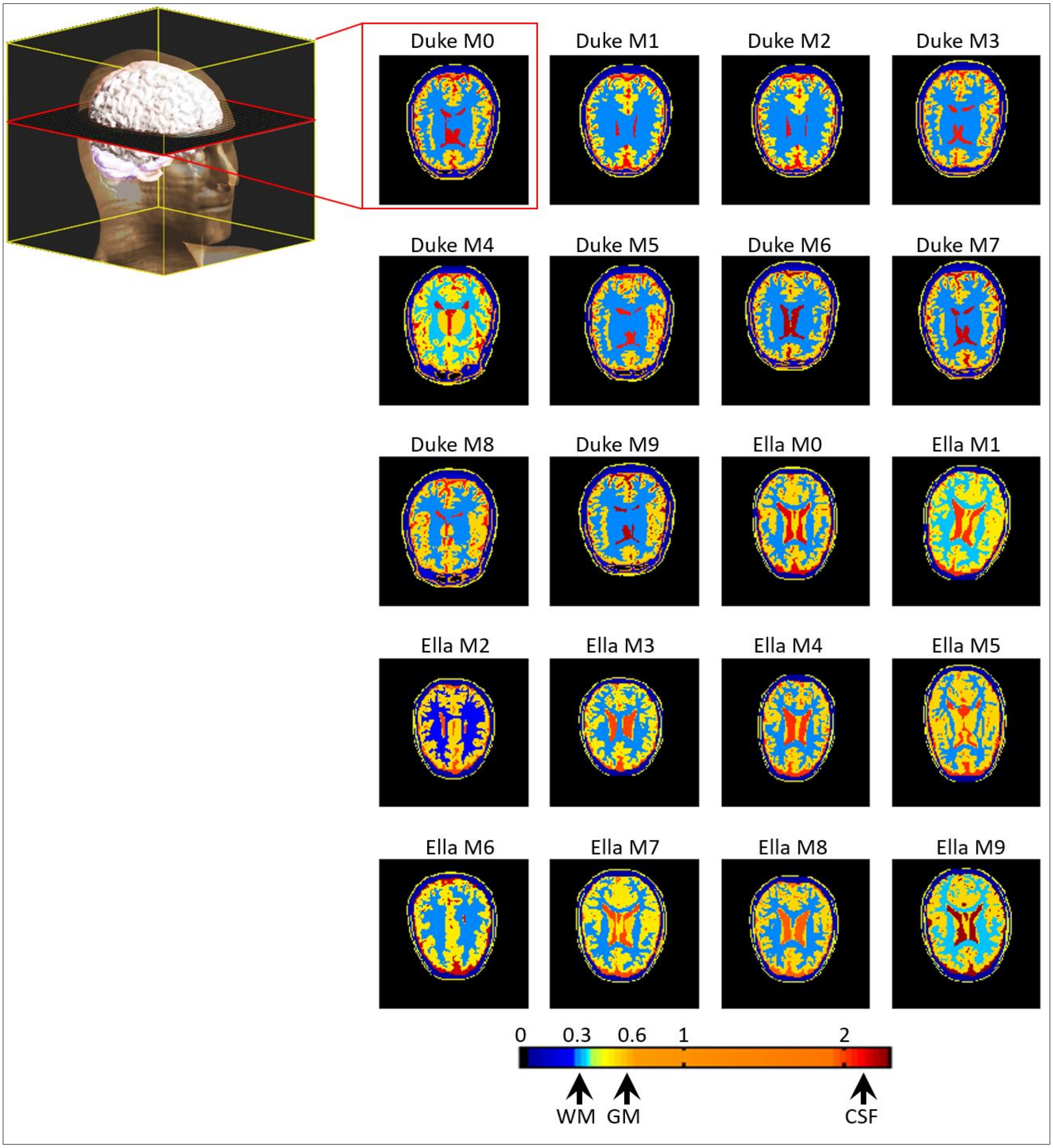

Supplementary Figure S1: Conductivity maps of the simulated head models. These maps were taken on the same slice (red plane) inside the considered volume of interest (yellow box).

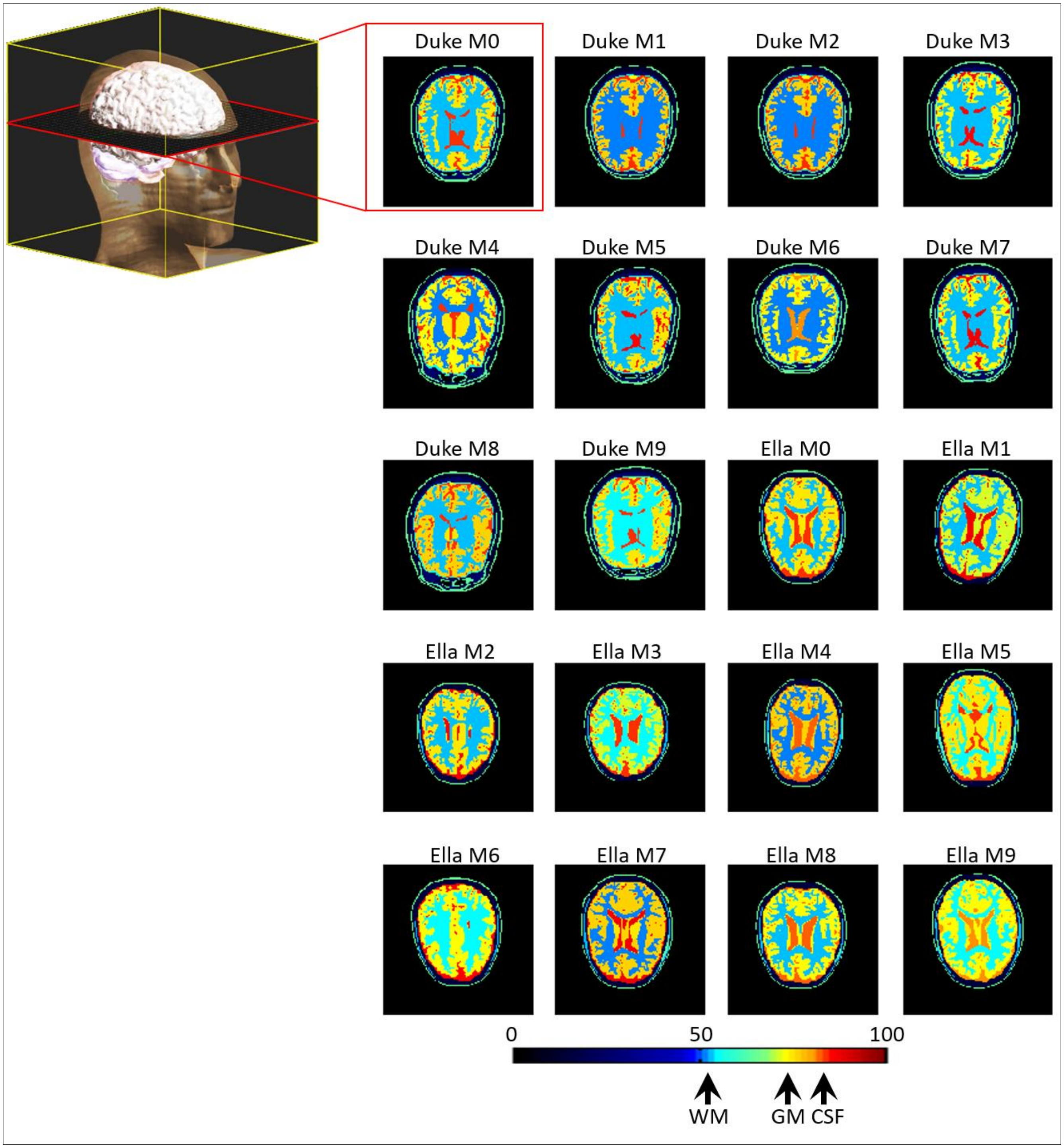

Supplementary Figure S2: Permittivity maps of the simulated head models. These maps were taken on the same slice (red plane) inside the considered volume of interest (yellow box).

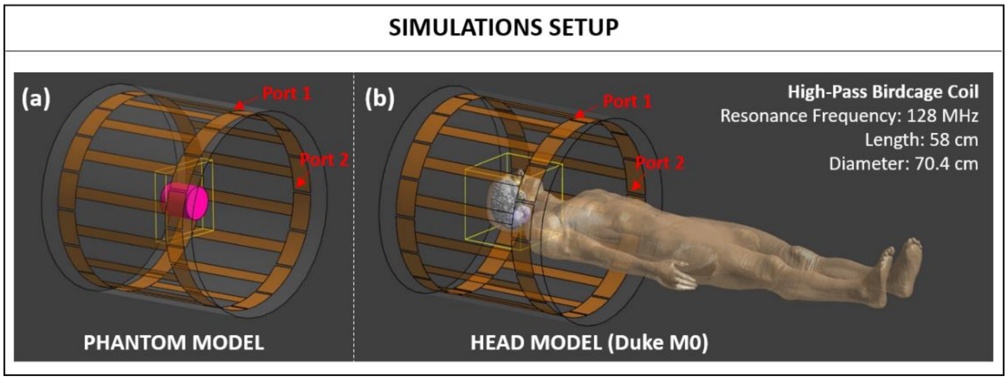

Supplementary Figure S3: The setup adopted in Sim4Life for the electromagnetic simulations on: (**a**) phantoms, (**b**) head models.

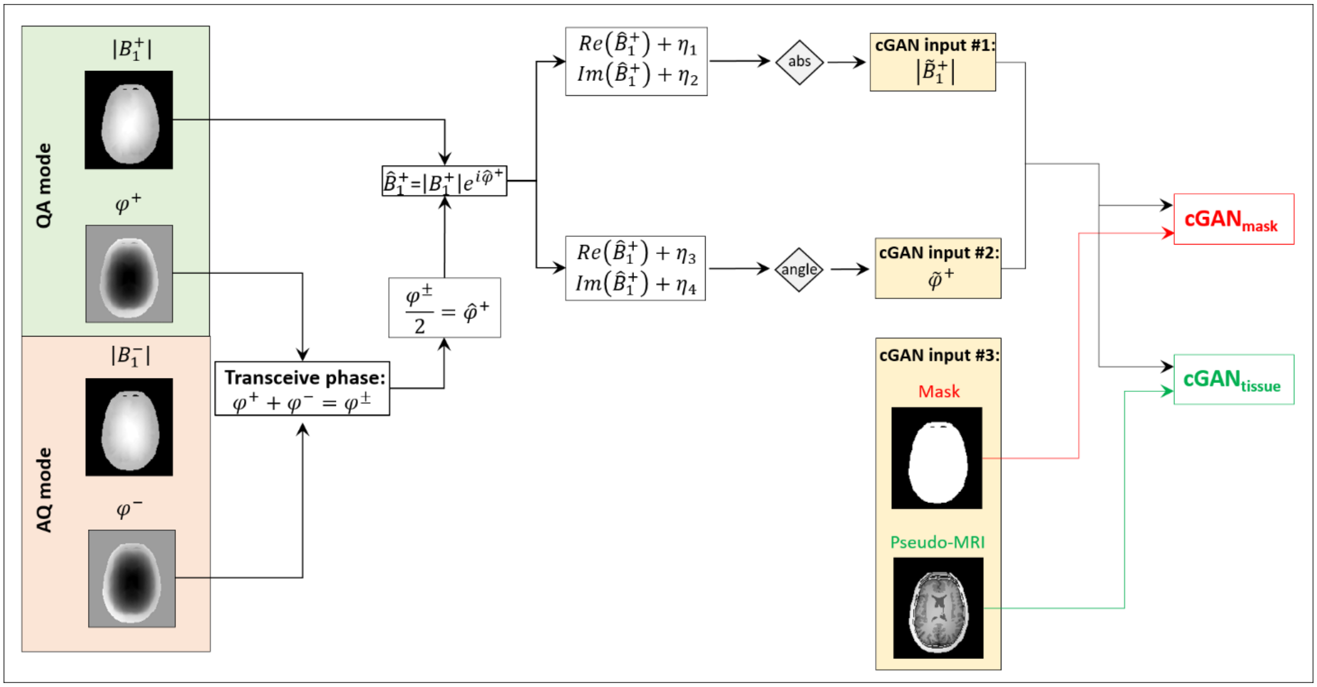

Supplementary Figure S4: Flowchart of the operations performed to create the input maps for the cGANs.

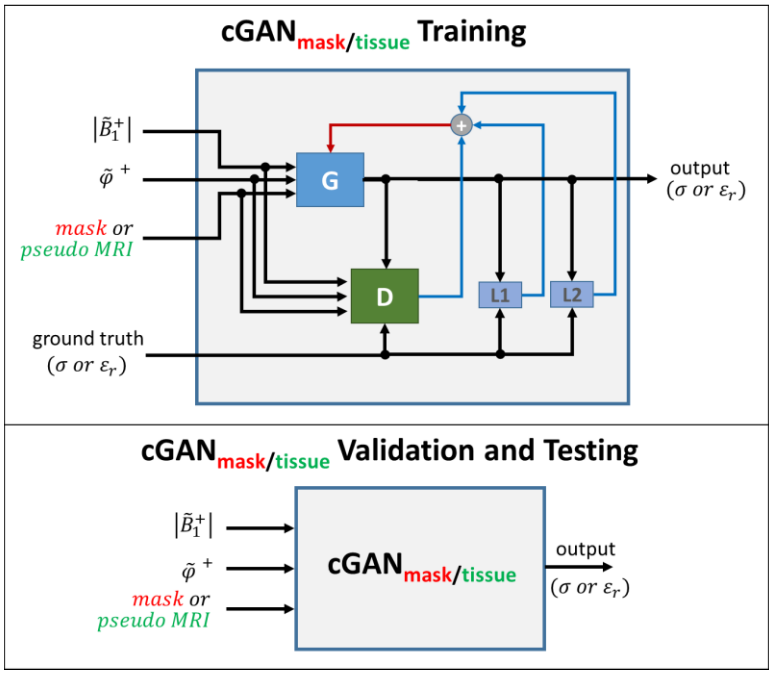

Supplementary Figure S5: Flowchart of the inputs/outputs of the adopted cGANs ($cGAN_{mask}$, and $cGAN_{tissue}$) for training, validation, and testing.

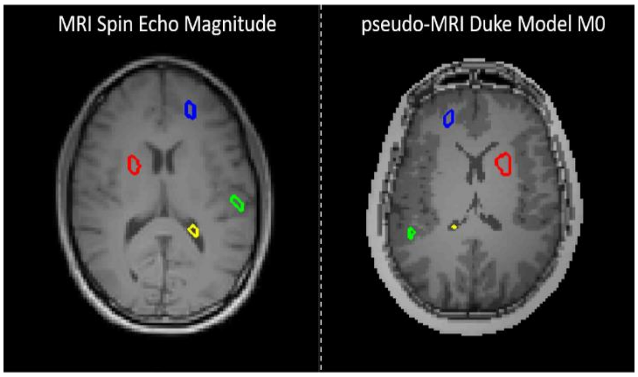

Supplementary Figure S6: Measured Spin Echo magnitude map (left) and pseudo Spin Echo map (right). The depicted four ROIs are used to compute the mean signal intensity values (see Supplementary Table S3). These maps were normalized between 0 and 1.

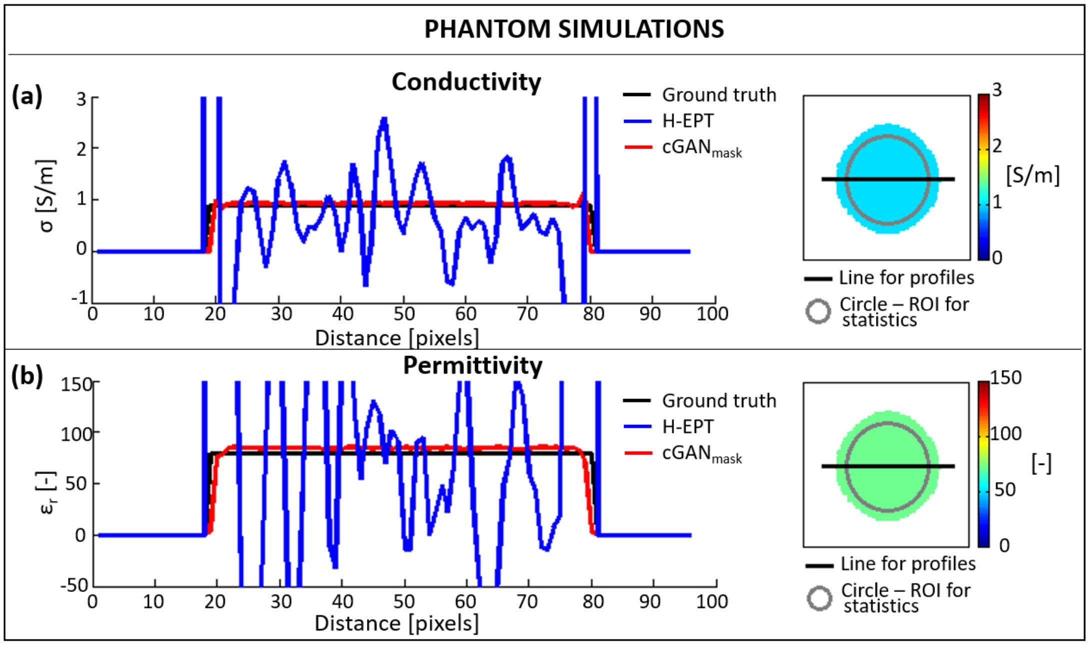

Supplementary Figure S7: Phantom model 42: profiles of the reconstructed EPs maps and definition of the region of interest (ROI) used to compute mean and SD of the reconstructed EPs values. These profiles show how the $cGAN_{mask}$ preserves boundaries better than H-EPT reconstructions.

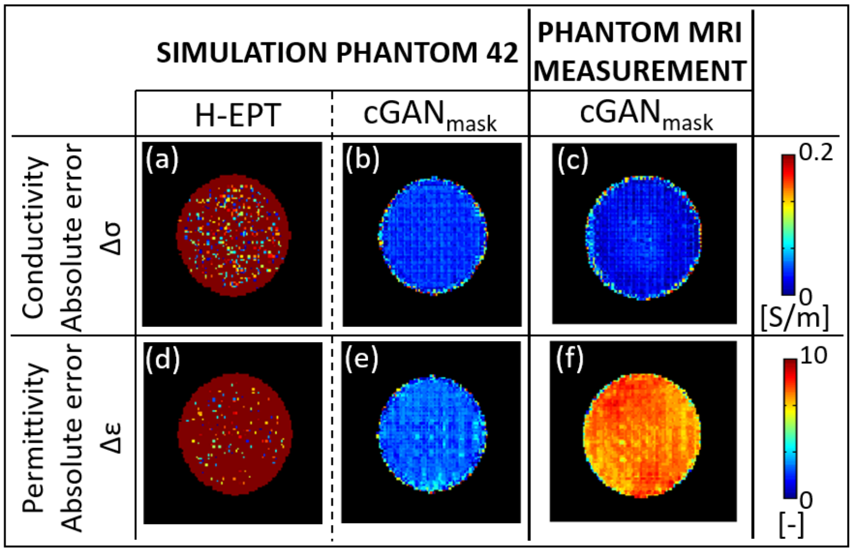

Supplementary Figure S8: Phantom model 42: absolute error maps for the reconstructed conductivity (**a**, **b**) and permittivity (**d**, **e**) maps using H-EPT (**a**, **d**) and $cGAN_{mask}$ (**b**, **e**). Phantom MRI measurements: absolute error maps for the reconstructed conductivity (**c**) and permittivity (**f**) maps using $cGAN_{mask}$.

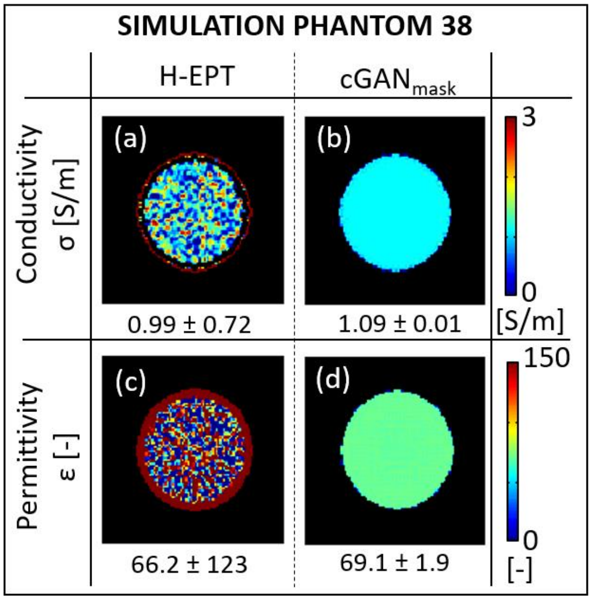

Supplementary Figure S9: Phantom 38 conductivity (**a**, **b**,) and permittivity (**c**, **d**,) maps reconstructed using H-EPT (**a**, **c**,) and $cGAN_{mask}$ (**b**, **d**,). The reported numbers are the mean ± SD values computed inside the region of interest indicated in the Supplementary Figure S7. Ground truth EPs values are respectively σ = 1 S/m and ε_r = 66 (see Table S1).

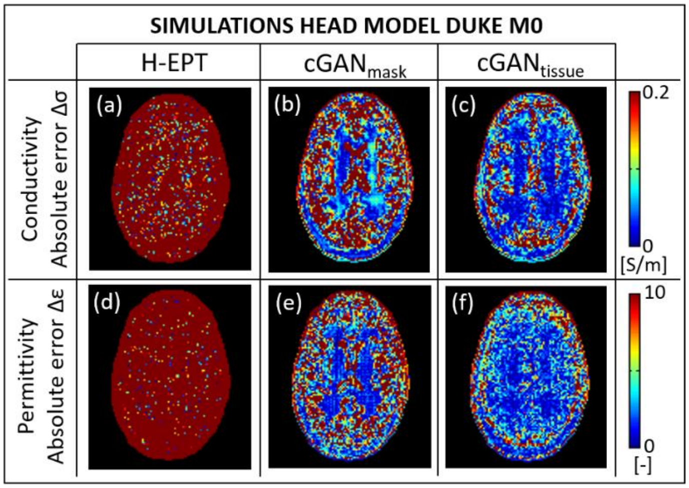

Supplementary Figure S10: Head model Duke M0: absolute error for the reconstructed conductivity (**a**, **b,** and **c**) and permittivity (**d**, **e**, and **f**) maps using H-EPT (**a**, **d**) and $cGAN_{mask}$ (**b**, **e**), and $cGAN_{tissue}$ (**c**, **f**).

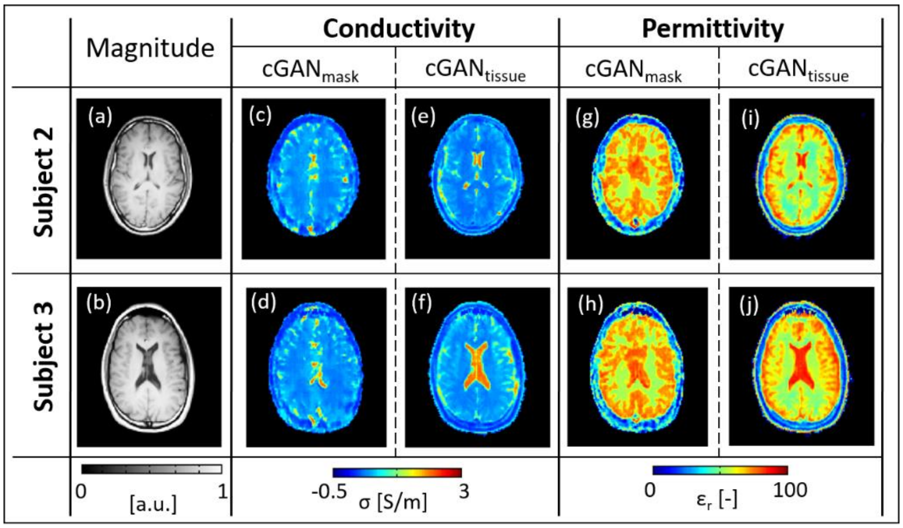

Supplementary Figure S11: DL-EPT reconstructions for the second and the third subject: reference Spin Echo magnitude images (**a**, **b**), reconstructed conductivity (**c**, **d**, **e**, and **f**) and permittivity (**g**, **h**, **i**, and **j**) maps using $cGAN_{mask}$ (**c**, **d**, **g**, and **h**), and $cGAN_{tissue}$ (**e**, **f**, **i**, and **j**).

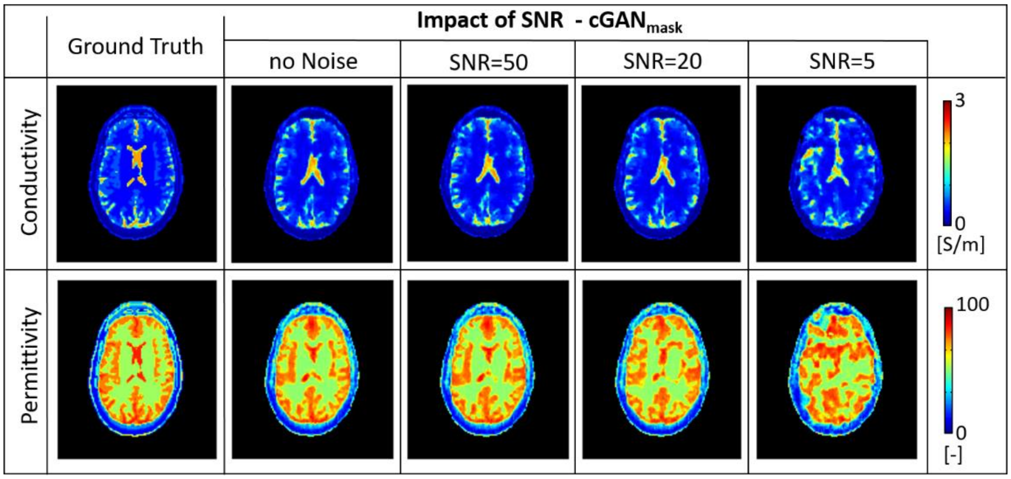

Supplementary Figure S12: $cGAN_{mask}$ EPs reconstructions using different SNR levels for Duke Model M0.

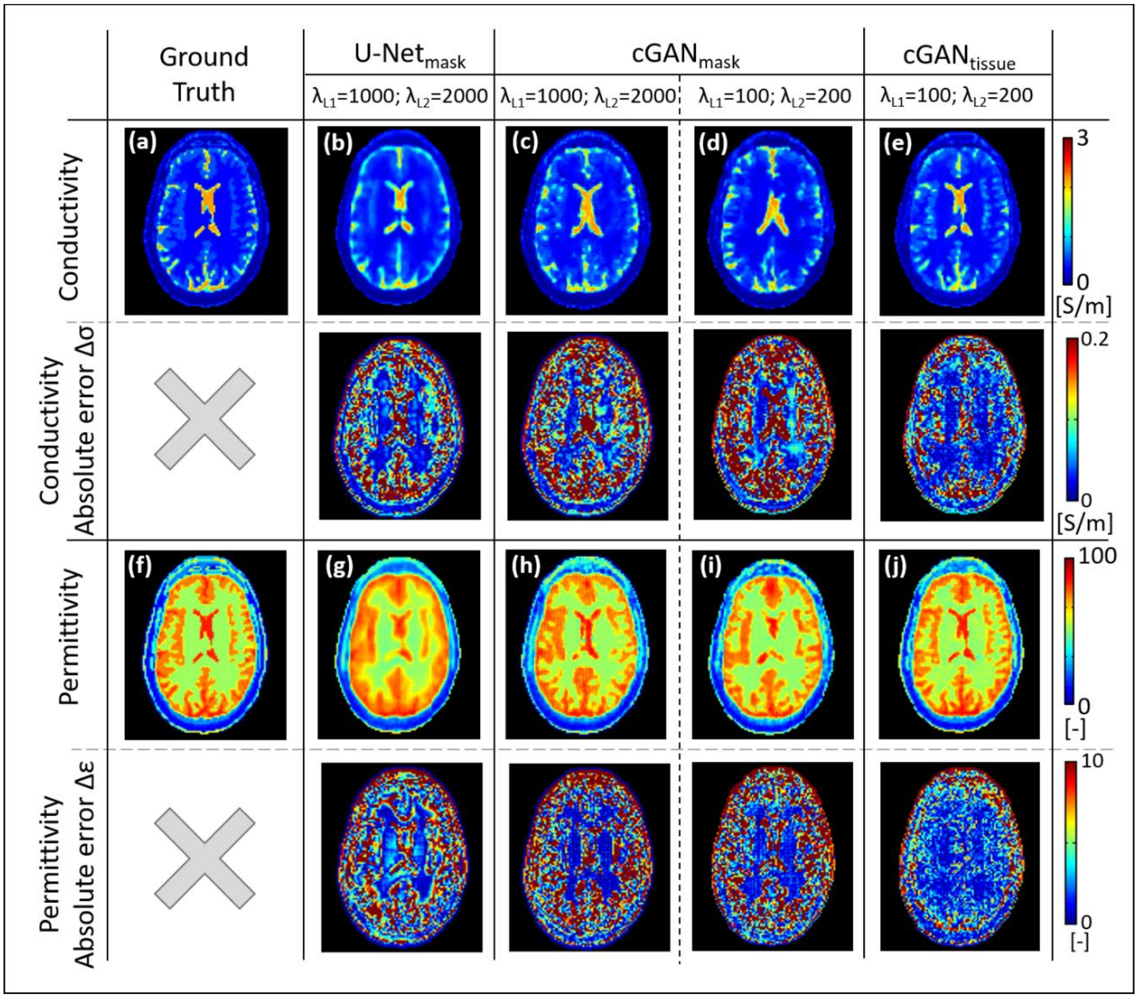

Supplementary Figure S13: Comparison between EPs reconstructions using a U-Net (**b**, **g**), the $cGAN_{mask}$ adopted for the tumor reconstruction from simulations using Duke M0 (**c**, **h**), and the $cGAN_{mask}$ (**d**, **i**) and $cGAN_{tissue}$ (**e**, **j**) adopted for DL-EPT reconstructions in the manuscript, i.e. for the phantom model 42, Duke M0, phantom and *in-vivo* brain MR measurements.

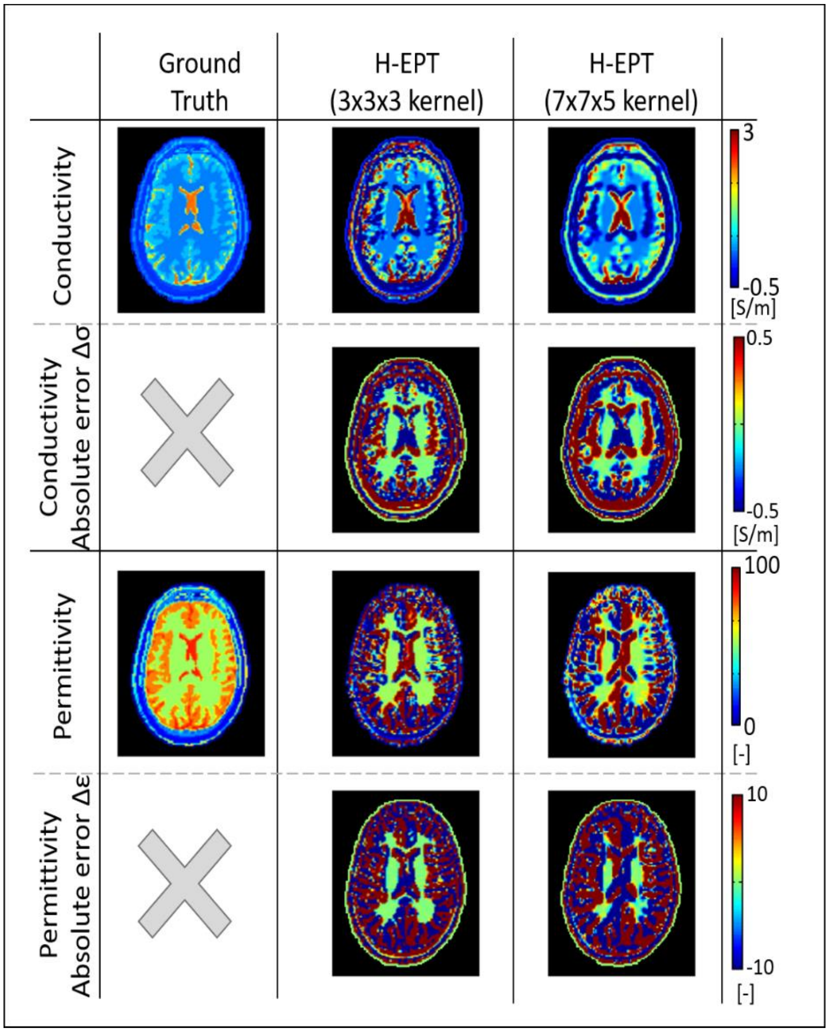

Supplementary Figure S14: Comparison between H-EPT reconstructions using a small kernel (3×3×3) and a large kernel (7×7×5) for the noiseless case. Notable the errors at tissue boundaries, which spatial extension increases for the large kernel. EPs reconstructions are accurate only inside large homogeneous regions of WM.

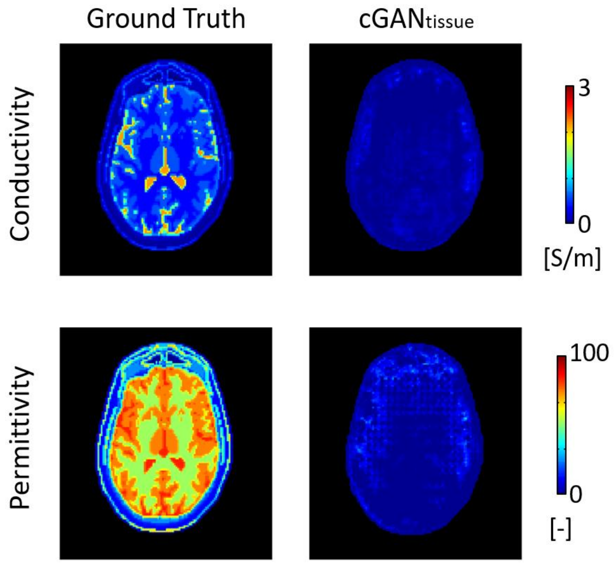

Supplementary Figure S15: $cGAN_{tissue}$ reconstructions given only the pseudo Spin Echo images of Duke M0 as input to the network.

Supplementary Table S1: Phantoms EPs values

PHANTOM	σ [S/m]	ε_r [-]
1	1.20	81
2	1.25	70
3	1.30	60
4	1.35	65
5	1.40	75
6	1.45	85
7	1.50	72
8	1.55	82
9	1.60	62
10	1.65	83
11	1.70	73
12	1.75	63
13	1.80	88
14	1.85	68
15	1.90	78
16	1.95	86
17	2.00	66
18	2.05	76
19	2.10	87
20	2.15	67
21	2.20	77
22	0.20	80
23	0.25	70
24	0.30	60
25	0.35	65
26	0.40	75
27	0.45	85
28	0.50	72
29	0.55	82
30	0.60	62
31	0.65	83
32	0.70	73
33	0.75	63
34	0.80	88
35	0.85	68
36	0.90	78
37	0.95	86
38	1.00	66
39	1.05	76
40	1.10	87
41	1.15	67
42	0.88	80

The models 12 and 24 are used for validation, while the models 38 and 42 are used for testing.

Supplementary Table S2. Head Models – Dimensional Scaling Factors and EPs values

		T_x	T_y	T_z	WM		GM		CSF	
		(%)	(%)	(%)	σ [S/m]	ε_r [-]	σ [S/m]	ε_r [-]	σ [S/m]	ε_r [-]
Duke	M0	100	100	100	0.34	52.6	0.59	73.4	2.14	84
	M1	101	100	100.5	0.35	50.5	0.56	75.5	2.10	83.0
	M2	101.5	101	100	0.35	51.0	0.57	72.5	2.18	84.5
	M3	100	101	102	0.33	52.0	0.58	73.0	2.08	85.0
	M4	101	102	102	0.36	51.0	0.56	73.2	2.15	83.5
	M5	95	100	106	0.35	53.0	0.59	74.0	2.05	84.6
	M6	102	94	92	0.33	51.5	0.60	73.0	2.20	81.0
	M7	94	102	100	0.34	53.0	0.60	72.0	2.16	86.0
	M8	102	102	94	0.35	52.0	0.59	75.0	2.06	82.5
	M9	103	96	103	0.35	53.4	0.60	74.7	2.21	84.0
Ella	M0	100	100	10	0.34	52.5	0.59	73.5	2.14	84.0
	M1	104	102	10	0.36	51.8	0.57	71.4	2.02	86.0
	M2	94	96	10	0.32	52.0	0.60	74.0	2.00	86.5
	M3	90	98	102	0.35	54.0	0.56	71.3	1.98	83.0
	M4	97	90	100	0.32	51.2	0.60	75.1	2.03	82.6
	M5	105	97	94	0.33	53.2	0.60	74.4	2.04	84.0
	M6	100	104	104	0.35	53.4	0.57	72.8	2.17	85.3
	M7	100	106	98	0.35	50.6	0.57	75.2	2.01	86.2
	M8	96	104	92	0.33	51.6	0.61	72.3	1.96	82.6
	M9	102	106	96	0.36	54.3	0.59	72.5	2.23	80.3

The electrical properties values of the 20 head models. T_x, T_y, and T_z are the scaling factors applied to the original models (M0) along the coordinate axis x, y, and z ($T_{x,y,z}$ = 100: no scaling, $T_{x,y,z}$ > 100: dilatation, and $T_{x,y,z}$ < 100: compression). The models Duke M0 and Ella M0 are the reference models.

Supplementary Table S3. Spin Echo Magnitude

ROI	MRI Spin Echo Magnitude	Pseudo Spin Echo Duke M0
Red (WM)	0.51	0.52
Blue (WM)	0.43	0.45
Green (GM)	0.39	0.36
Yellow (CSF)	0.25	0.27

Comparison between measured and pseudo Spin Echo magnitude values in the four ROIs depicted in Supplementary Figure S6.

Supplementary Table S4. Choice of $cGAN_{mask}$ trained with different parameters

$cGAN_{mask}$ parameters			Phantom 12		Phantom 24		Average NRMSE [%]
λ_{GAN}	λ_{L1}	λ_{L2}	σ [S/m] mean (SD)	ε_r [-] mean (SD)	σ [S/m] mean (SD)	ε_r [-] mean (SD)	
0	1000	2000	1.88 (0.02)	75.2 (2.1)	0.33 (0.07)	66.1 (1.1)	11.8
2	100	0	1.97 (0.02)	72.7 (2.5)	0.33 (0.02)	65.5 (0.4)	12.5
2	100	200	1.85 (0.02)	65.6 (2.4)	0.28 (0.02)	66.4 (0.4)	7.8
2	1000	0	1.90 (0.01)	73.5 (1.9)	0.30 (0.02)	65.4 (0.5)	9.9
2	1000	1000	1.95 (0.01)	71.4 (4.1)	0.28 (0.01)	64.1 (0.6)	9.7
2	1000	2000	1.86 (0.02)	74.6 (1.9)	0.33 (0.01)	72.1 (0.9)	14.4
Reference EPs values			*1.75 (-)*	*63 (-)*	*0.3 (-)*	*60 (-)*	-

Mean EPs values and SD (between brackets) of the two phantoms used for the validation of the trained $cGAN_{mask}$. The percentage of the average NRMSE computed over the reconstructed EPs values of both phantoms is reported in the last column.

Supplementary Table S5. AFI Sequence Parameters

AFI	TR1	TR2	TE	Flip Angle	Field of View	Voxel size
Phantom	50 ms	250 ms	2.5 ms	65°	$256\times256\times75$ mm^3	$2\times2\times3$ mm^3
In-vivo	50 ms	250 ms	2.5 ms	65°	$256\times256\times90$ mm^3	$2\times2\times3$ mm^3

Sequence parameters used for the AFI sequence. This sequence was adopted to map the magnitude of the transmit MR field.

Supplementary Table S6. Spin Echo Sequence Parameters

Spin Echo	TR	TE	Field of View	Voxel size
Phantom	900 ms	5 ms	$256\times256\times75$ mm^3	$2\times2\times3$ mm^3
In-vivo	900 ms	5 ms	$256\times256\times90$ mm^3	$2\times2\times3$ mm^3

For both phantom and *in-vivo* MR measurements, this sequence was performed twice, i.e. with opposite readout gradient polarities to compensate for eddy-currents related artifacts. This sequence was adopted to map the transceive phase.

Supplementary Table S7. ***In-vivo*** **DL-EPT reconstructions**

		WM		GM		CSF	
		**σ [S/m] mean (SD)**	**ε_r [-] mean (SD)**	**σ [S/m] mean (SD)**	**ε_r [-] mean (SD)**	**σ [S/m] mean (SD)**	**ε_r [-] mean (SD)**
Subject 2	$cGAN_{mask}$	0.41 (0.09)	63.2 (8.6)	0.62 (0.31)	71.7 (4.6)	1.09 (0.59)	76.2 (6.4)
	$cGAN_{tissue}$	0.32 (0.04)	49.5 (2.7)	0.45 (0.05)	60.6 (4.1)	1.87 (0.45)	82.4 (4.8)
Subject 3	$cGAN_{mask}$	0.38 (0.12)	55.8 (6.1)	0.48 (0.12)	67.5 (7.5)	0.76 (0.47)	74.2 (6.9)
	$cGAN_{tissue}$	0.39 (0.04)	54.2 (2.0)	0.52 (0.12)	65.8 (5.7)	2.05 (0.20)	83.7 (1.7)
reference		*0.34 (-)*	*52.6 (-)*	*0.59 (-)*	*73.4 (-)*	*2.14 (-)*	*84 (-)*

Mean and SD (inside brackets) of the reconstructed EPs values in the WM, GM, and CSF tissues from *in-vivo* MR measurements for the second and the third subject.

Supplementary Table S8. EPs Reconstructions for different SNR levels using $cGAN_{mask}$ and Duke M0

		WM		GM		CSF	
$\mathbf{SNR}_{\lvert\tilde{B}_1^+\rvert}$	$\Delta\tilde{\varphi}^+$	**σ [S/m] mean (SD)**	**ε_r [-] mean (SD)**	**σ [S/m] mean (SD)**	**ε_r [-] mean (SD)**	**σ [S/m] mean (SD)**	**ε_r [-] mean (SD)**
No-noise	No-noise	0.38 (0.19)	54.9 (6.9)	0.65 (0.35)	71.9 (7.9)	1.77 (0.51)	83.1 (4.5)
50	0.02	0.38 (0.19)	55.1 (7.1)	0.65 (0.35)	71.7 (8.1)	1.77 (0.52)	82.9 (4.6)
20	0.05	0.38 (0.20)	56.1 (7.8)	0.65 (0.35)	71.3 (8.6)	1.76 (0.52)	82.2 (5.3)
5	0.2	0.44 (0.26)	62.5 (11.1)	0.67 (0.39)	68.9 (11.3)	1.58 (0.59)	78.7 (8.3)
reference		*0.34 (-)*	*52.6 (-)*	*0.59 (-)*	*73.4 (-)*	*2.14 (-)*	*84 (-)*

Mean values and SD (inside brackets) of the reconstructed EPs in the WM, GM, and CSF tissues for the head model Duke M0 using $cGAN_{mask}$ and different SNR levels. To exclude numerical errors at tissue boundaries that might arise from discretization and resizing of the simulated electromagnetic fields, a 1 voxel erosion was performed for each tissue type.

Supplementary Table S9. **EPs Reconstructions for Duke M0 using $cGAN_{mask}$ trained with different parameters**

$cGAN_{mask}$ parameters			WM		GM		CSF		Average NRMSE [%]
λ_{GAN}	λ_{L1}	λ_{L2}	σ [S/m] mean (SD)	ε_r [-] mean (SD)	σ [S/m] mean (SD)	ε_r [-] mean (SD)	σ [S/m] mean (SD)	ε_r [-] mean (SD)	
0	1000	2000	0.35 (0.06)	55.2 (4.7)	0.62 (0.15)	73.3 (3.8)	2.06 (0.19)	83.2 (2.7)	11.8
2	100	0	0.41 (0.15)	55.6 (5.4)	0.67 (0.30)	71.8 (6.7)	1.90 (0.37)	82.9 (4.7)	24.8
2	100	200	0.38 (0.19)	54.9 (7.0)	0.65 (0.35)	71.9 (7.9)	1.77 (0.51)	83.0 (4.5)	29.4
2	1000	0	0.42 (0.15)	55.3 (6.5)	0.65 (0.30)	71.1 (6.9)	1.83 (0.47)	80.9 (4.9)	26.6
2	1000	1000	0.42 (0.17)	55.7 (6.4)	0.66 (0.34)	69.6 (6.0)	1.89 (0.44)	79.6 (5.8)	28.3
2	1000	2000	0.39 (0.14)	54.8 (5.7)	0.66 (0.30)	72.1 (6.7)	1.97 (0.33)	81.8 (3.9)	23.3
reference			*0.34 (-)*	*52.6 (-)*	*0.59 (-)*	*73.4 (-)*	*2.14 (-)*	*84 (-)*	-

Mean and the SD (inside brackets) of the reconstructed EPs values in the WM, GM, and CSF for Duke M0 are reported for different $cGAN_{mask}$ parameters combinations (λ_{GAN}, λ_{L1}, and λ_{L2}). In the last column, the percentage of the average NRMSE among the reconstructed EPs values in these three tissues is reported for each parameters combination. To exclude numerical errors at tissue boundaries that might arise from discretization and resizing of the simulated electromagnetic fields, a 1 voxel erosion was performed for each tissue type.
